# Prospective network analysis of proinflammatory proteins, lipid markers, and depression components in midlife community women

**DOI:** 10.1017/S003329172200232X

**Published:** 2023-08

**Authors:** Nur Hani Zainal, Michelle G. Newman

**Affiliations:** 1 Department of Health Care Policy, Harvard Medical School, Boston, MA, USA; 2 Department of Psychology, The Pennsylvania State University, State College, PA, USA

**Keywords:** Cross-lagged, depression, endocrine, immune, inflammation, interpersonal, network analysis, scar theory, vulnerability theory

## Abstract

**Background:**

Vulnerability theories propose that suboptimal levels of lipid markers and proinflammatory proteins predict future heightened depression. Scar models posit the reverse association. However, most studies that tested relationships between non-specific immune/endocrine markers and depression did not separate temporal inferences between people and within-person and how different immunometabolism markers related to unique depression symptoms. We thus used cross-lagged prospective network analyses (CLPN) to investigate this topic.

**Methods:**

Community midlife women (*n* = 2224) completed the Center for Epidemiologic Studies-Depression scale and provided biomarker samples across five time-points spanning 9 years. CLPN identified significant relations (*edges*) among components (*nodes*) of depression (depressed mood, somatic symptoms, interpersonal issues), lipid markers [insulin, fasting glucose, triglycerides, low-density lipoprotein-cholesterol (LDL), high-density lipoprotein-cholesterol (HDL)], and proinflammatory proteins [C-reactive protein (CRP), fibrinogen], within and across time-points. All models adjusted for age, estradiol, follicle-stimulating hormone, and menopausal status.

**Results:**

In within-person temporal networks, higher CRP and HDL predicted all three depression components (*d* = 0.131–2.112). Increased LDL preceded higher depressed mood and interpersonal issues (*v.* somatic symptoms) (*d* = 0.251–0.327). Elevated triglycerides predicted more somatic symptoms (*v.* depressed mood and interpersonal problems) (*d* = 0.131). More interpersonal problems forecasted elevated fibrinogen and LDL levels (*d* = 0.129–0.331), and stronger somatic symptoms preceded higher fibrinogen levels (*d* = 0.188).

**Conclusions:**

Results supported both vulnerability and scar models. Long-term dysregulated immunometabolism systems, social disengagement, and related patterns are possible mechanistic accounts. Cognitive-behavioral therapies that optimize nutrition and physical activity may effectively target depression.

Heightened depression symptoms are commonly observed in the general population annually and across the lifetime (Jeuring et al., 2018). Reliable evidence has linked subthreshold depression to many physical ailments involving the cardiometabolic, gastrointestinal, and autoimmune systems (Simpson et al., 2021). Elevated depression also adversely affects romantic and professional relationships, career development, and other life satisfaction domains (Sivertsen, Bjorklof, Engedal, Selbaek, & Helvik, 2015). Economically, heightened depression consumes significant annual government expenditure (Revicki et al., 2012). Thus, a better understanding of the risk factors and consequences of elevated depression components is essential.

Our immune and endocrine systems dynamically interact with depressed mood and related symptoms by regulating the sympathetic nervous system, vagus nerve, hypothalamic–pituitary axis (HPA), and associated systems (Peirce & Alvina, 2019; Thayer & Fischer, 2009). These regulatory systems optimize inflammation levels to fend off infections, injuries, and toxins (Ellins, Rees, Deanfield, Steptoe, & Halcox, 2017). Two types of inflammation exist. Short-term (acute) inflammation is triggered by sugary and fatty substances, viruses, and bacteria that may result from sickness recovery, wound reparation, and brief stress episodes and is, on balance, adaptive (Cecconello, Clària Ribas, & Norling, 2022). Conversely, long-term (chronic) inflammation can build up plaques, clot the bloodstream, and impair the brain, heart, and other organs (Michels, van Aart, Morisse, Mullee, & Huybrechts, 2021). Likewise, our endocrine system, which comprises glands that secrete and absorb hormones, lipids, and related markers, needs optimal balance to modulate mood states effectively (Chen et al., 2016). Prolonged inflammation and suboptimal lipid marker levels can thus contribute to autoimmune disorders, depressed mood, and associated symptoms by lowering resilience to stress and corresponding processes (Dedoncker, Vanderhasselt, Ottaviani, & Slavich, 2021; Suvarna et al., 2020).

Potential risk factors or consequences of elevated depression components have been theorized to include suboptimal levels of chronic peripheral proinflammatory proteins and lipid markers (e.g. Penninx, 2017). Proxy lipid markers might comprise unique hormones (e.g. insulin, fasting glucose), fats (e.g. triglycerides), and a combination of proteins and fats [e.g. low-density lipoprotein cholesterol (LDL), high-density lipoprotein cholesterol (HDL)] (Marz et al., 2017). C-reactive protein (CRP) and fibrinogen are acute-phase proinflammatory proteins the liver secretes in response to increased interleukin-6 (IL-6) and tend to be embedded in plasma and other bodily fluids (Johansson-Persson et al., 2014). CRP, in conjunction with damaged cells or sets of disease-producing microorganisms, primarily serve to activate adjunct systems (Macleod & Avery, 1941). Fibrinogen is a clotting agent precursor of the enzyme fibrin and is instrumental in platelet aggregation when fixing tissue and vascular injuries but contributes to heart problems in excessive amounts (Duivis et al., 2011). Depression components include somatic symptoms (e.g. appetite changes, sleep disturbances), depressed mood, and interpersonal problems (e.g. perceived unfriendliness) (Cosco, Prina, Stubbs, & Wu, 2017). These variables have been incorporated into vulnerability and scar theories of depression.

In particular, *vulnerability models* propose that suboptimal levels of insulin, fasting glucose, triglycerides, LDL, and HDL might influence future somatic (*v.* mood and interpersonal) aspects of depression (Lamers et al., 2020; Penninx, 2017). Surrogate lipid markers have been theorized to predict future depressed mood and somatic symptoms through reduced neurogenesis, suboptimal cell metabolism function, and heightened inflammation (Dantzer, O'Connor, Freund, Johnson, & Kelley, 2008). These processes could decrease motivation to engage regularly in healthy behaviors and physical activity (Ignacio, da Silva, Plissari, Quevedo, & Reus, 2019), leading to subsequent depressed mood. Proinflammatory proteins that likely predict multiple aspects of depression include CRP and fibrinogen (Konsman, 2019; Lafitte et al., 2015). As they deplete dopaminergic neurons and disrupt mitochondrial function (e.g. glucose production) (Dantzer, Casaril, & Vichaya, 2021), increased proinflammatory proteins (*v.* proxy lipid markers) would likely more strongly impact somatic (*v.* mood and interpersonal) depression components (Majd, Saunders, & Engeland, 2020). Proinflammatory proteins (*v.* proxy lipid markers) could to a larger degree, perpetuate ‘sickness behaviors’ (i.e. fatigue, reduced activities) and negatively impact emotion regulation-related brain areas (e.g. dorsal anterior cingulate cortex, ventromedial prefrontal cortex) (Felger et al., 2016; Torres-Platas, Cruceanu, Chen, Turecki, & Mechawar, 2014), leading to future-elevated somatic symptoms.

*Scar theories* posit that somatic symptoms, compared to depressed mood and interpersonal issues, are depression components with the most extensive relations to future increased proinflammatory proteins (Felger et al., 2020; Lamers et al., 2013) *v.* surrogate lipid markers (Rotella & Mannucci, 2013). These processes could occur via the buildup of stress hormones and chronic dysregulation of the HPA over long periods (Dias et al., 2020; Vingeliene, Hiyoshi, Lentjes, Fall, & Montgomery, 2019). Suboptimal habits (e.g. decreased exercise, excessive caloric intake, or carbohydrate-dense foods) and social withdrawal patterns (Feng & Astell-Burt, 2017) could mediate elevated depression components predicting worse immunometabolism. Also, depression components might adversely affect immunometabolism via decreased attempts to tap into social support resources during stress (Gouin, Wrosch, McGrath, & Booij, 2020). Increased social isolation could negatively alter the body's reactivity toward biological or interpersonal stressors (e.g. worsening social cohesion) (Smith, Gavey, NE, Kontari, & Victor, 2020). These challenges could prompt more robust long-term increased proinflammatory (*v.* proxy lipid markers) responses, resulting in more somatic symptoms relative to depressed mood and interpersonal issues (Smith et al., 2020).

Prospective data to date reliably support the theories above. Consistent with vulnerability and scar models, data across 15 studies showed that excessive surrogate lipid markers (e.g. insulin, fasting glucose) bidirectionally predicted future major depression severity and diagnosis in clinical and community samples (cf. meta-analyses and empirical study by Hiles, Revesz, Lamers, Giltay, and Penninx, 2016; Pan et al., 2012). Likewise, concordant with vulnerability models and scar theories, more than 85 studies that recruited diverse youth and adult populations showed that depressive symptoms bidirectionally predicted heightened surrogate lipid markers and non-specific proinflammatory proteins (e.g. IL-6, CRP, fibrinogen) across 2 months to 18 years (cf. reviews and empirical studies by Colasanto, Madigan, and Korczak, 2020; Lamers et al., 2019; Mac Giollabhui, Ng, Ellman, and Alloy, 2021; Valkanova, Ebmeier, and Allan, 2013; Zainal and Newman, 2021c, 2022). Collectively, suboptimal proinflammatory proteins and surrogate lipid marker levels could be bidirectionally related to somatic symptoms, depressed mood, and interpersonal problems. Moreover, the literature offers more evidence for vulnerability models than for scar theories (e.g. Mac Giollabhui et al., 2021).

However, most prior longitudinal studies thus far have not tested how *components* of proinflammatory proteins, surrogate lipid markers, and depression related to one another. Examining these relationships is essential because depression may arise from the interactions among these mutually influencing components, and unique depression components could relate differently in magnitude and direction to distinct surrogate immunometabolism markers (Zhang et al., 2022). In addition, the literature is replete with studies on this topic using ordinary least squares (OLS) regression and structural equation modeling (SEM) approaches. Although informative, OLS, SEM, and other traditional statistical approaches tend to yield parameters that enable understanding of the relations among the *mean-overall score or latent constructs* rather than relations among the *components* of these constructs. The latent variable modeling approach precludes determining unique immunometabolism trajectories for persons with the same mean-overall score but elevated on different components (e.g. high somatic symptoms and low depressed mood *v.* low somatic symptoms and high depressed mood). Cross-lagged prospective network analysis (CLPN) (Epskamp, 2020) is thus a means to understand how components (or *nodes*) rather than latent constructs relate to one another in a network of mutually influencing nodes across multiple time-points within and between persons. Relations between nodes are called *edges*, typically expressed as partial correlations that have adjusted for the effects of all other nodes. Moreover, CLPN permits identifying nodes with the biggest impact and the highest number of associations with all future nodes (Borsboom et al., 2021). These most impactful nodes in temporal networks are key therapy targets, as altering those influential nodes might change future depression nodes (Roefs et al., 2022).

To date, only six studies have used network analyses with cross-sectional data to investigate this topic. Recently, Jia et al. (2020) observed that although higher HDL levels coincided with stronger concurrent depressive symptoms, other lipid markers (e.g. triglycerides, LDL) had null relations. Furthermore, depressive symptoms were nodes with the most robust connections with other nodes in the network (Jia et al., 2020). A separate network analysis showed that higher IL-6 and CRP more strongly coincided with increased somatic symptoms (i.e. aches, pains, sleep issues) *v.* other depression nodes in Dutch adults with and without elevated depression (Fried et al., 2020). Another network analysis found that persons with (*v.* without) heightened CRP had more notable edges in a depression network, with thicker networks indicating more significant psychopathology (Moriarity, van Borkulo, & Alloy, 2021b). Concentration deficits and psychomotor problems (*v.* other depression nodes) were the most influential in this study (Moriarity et al., 2021b). Moreover, higher CRP showed the largest associations with appetite changes and fatigue than other depression nodes in another large community sample (Moriarity, Horn, Kautz, Haslbeck, & Alloy, 2021a). Likewise, the polygenic risk score of CRP (but not IL-6 and other proinflammatory proteins) was most potently linked to fatigue and decreased anhedonia (Kappelmann et al., 2021). In addition, levels of triglyceride, total cholesterol, and insulin resistance, but not HDL, displayed the most substantial concurrent relations with higher depression severity in Korean adults (Nam, Peterson, Seo, Han, & Kang, 2021).

Therefore, the current study used CLPN to better understand the relations among surrogate lipid markers, proinflammatory proteins, and depression nodes across five time-points spanning 9 years. This research aim is essential for multiple reasons. Globally, metabolic syndrome-linked disorders (e.g. diabetes) and depressive disorders have increased (Jeuring et al., 2018; Leon & Maddox, 2015). Enhancing knowledge of the modifiable risk factors and outcomes for depression and related immunometabolism problems can facilitate fine-tuning current evidence-based treatments (e.g. physical exercise-focused behavioral therapies; Li et al., 2017). Also, most studies examining the links among depression components, proinflammatory proteins, and surrogate lipid markers have been cross sectional (e.g. Persons and Fiedorowicz, 2016), hindering weak causal inferences (Blanchard, Contreras, Kalkan, & Heeren, in press). Thus, based on theory and evidence, we tested two hypotheses. First, we hypothesized that the within-person temporal (lag-1) network would show evidence more consistent with vulnerability models than scar theories (hypothesis 1). Second, we expected that within and between persons, somatic symptoms (*v.* depressed mood and interpersonal problems) would have stronger associations with levels of proinflammatory proteins (*v.* surrogate lipid markers) (hypothesis 2).

## Method

### Participants

The present study was a secondary analysis of merged open-access datasets from the Study of Women's Health Across the Nation (SWAN) project (Greendale et al., 2010). At wave 1 (W1), the all-female participants (*n* = 2224) had a mean age of 45.96 years (s.d. = 2.67, range = 42–53) (refer to Table 1). Table S1(a) in the online Supplementary material details the descriptive statistics of demographic and study variables with the non-imputed dataset. Online Supplementary Table S1(b) offers descriptive statistics on related variables not included in the final analyses.

**Table 1.** Descriptive statistics of network nodes across all waves for multiply imputed dataset

	Wave 1			Wave 2			Wave 4			Wave 6			Wave 8		
	*n*	*M*	(s.d.)	*n*	*M*	(s.d.)	*n*	*M*	(s.d.)	*n*	*M*	(s.d.)	*n*	*M*	(s.d.)
Age	2224	1.524	(0.627)	2224	1.495	(0.621)	2224	1.473	(0.600)	2224	1.466	(0.597)	2224	1.429	(0.588)
Age (unscaled)	2224	45.964	(2.674)	2224	46.996	(2.684)	2224	49.031	(2.694)	2224	51.049	(2.684)	2224	53.094	(2.689)
Depressed mood	2224	1.799	(0.612)	2224	1.785	(0.629)	2224	1.768	(0.618)	2224	1.781	(0.637)	2224	1.699	(0.584)
Somatic symptoms	2224	1.426	(0.621)	2224	1.390	(0.594)	2224	1.398	(0.599)	2224	1.365	(0.58)	2224	1.347	(0.577)
Interpersonal problems	2224	1.918	(0.321)	2224	1.922	(0.337)	2224	2.242	(0.252)	2224	2.072	(0.303)	2224	2.005	(0.424)
Fibrinogen	2224	1.103	(0.169)	2224	1.078	(0.148)	2224	1.307	(0.182)	2224	1.111	(0.185)	2224	1.197	(0.316)
CRP	2224	1.352	(0.234)	2224	1.286	(0.256)	2224	1.835	(0.226)	2224	1.528	(0.268)	2224	1.302	(0.217)
Glucose	2224	1.067	(0.096)	2224	1.066	(0.101)	2224	1.086	(0.077)	2224	1.136	(0.168)	2224	1.093	(0.130)
Insulin	2224	1.203	(0.197)	2224	1.190	(0.174)	2224	1.145	(0.158)	2224	1.203	(0.196)	2224	1.238	(0.167)
Triglycerides	2224	2.140	(0.403)	2224	2.156	(0.428)	2224	2.165	(0.483)	2224	2.215	(0.446)	2224	2.305	(0.481)
LDL	2224	2.056	(0.458)	2224	2.030	(0.457)	2224	2.251	(0.412)	2224	2.018	(0.386)	2224	2.084	(0.484)
HDL	2224	2.081	(0.729)	2224	2.081	(0.729)	2224	2.081	(0.729)	2224	2.081	(0.729)	2224	2.081	(0.729)
FSH	2224	1.256	(0.280)	2224	1.328	(0.367)	2224	1.343	(0.347)	2224	1.436	(0.367)	2224	1.535	(0.345)
Estradiol	2224	1.144	(0.168)	2224	1.239	(0.302)	2224	1.224	(0.280)	2224	1.047	(0.099)	2224	1.098	(0.199)
Menopausal stage	*n*	%		*n*	%		*n*	%		*n*	%		*n*	%	
Pre	1236	55.576	–	829	37.275	–	643	28.912	–	409	18.390	–	50	2.248	–
Early peri	988	44.424	–	1254	56.385	–	1078	48.471	–	805	36.196	–	576	25.899	–
Late peri	–	–	–	99	4.451	–	199	8.948	–	234	10.522	–	241	10.836	–
Post	–	–	–	42	1.888	–	304	13.669	–	776	34.892	–	1357	61.016	–

*Note*: *M*, mean; s.d., standard deviation; Min, minimum; Max, maximum.; LDL, low density lipoprotein; HDL, high density lipoprotein; CRP, C-reactive protein; FSH, follicle-stimulating hormone; Pre, pre-menopausal; Peri, peri-menopausal; Post, post-menopausal. All scores have been rescaled to range from 1 to 4.

### Procedures

Participants completed a depression self-report and biomarker data collection protocols at W1 (1997–1998), wave 2 (W2; 1998–2000), wave 3 (W3; 2000–2002), wave 4 (W4; 2002–2004), and wave 5 (W5; 2004–2006). These five time-points were selected as they contained data relevant to our research question. Both self-reports and biomarker assays were collected on the same day of the study visit (El Khoudary et al., 2016; McClure et al., 2014).

### Measures

#### Surrogate lipid markers

Ethylenediaminetetraacetic (EDTA)-treated plasma and enzymatic approaches determined the levels of triglycerides and LDL (Myers, Cooper, Winn, & Smith, 1989). Heparin-2M manganese chloride facilitated the extraction of HDL levels (Warnick & Albers, 1978). The radioimmunoassay (DPC Coat-a-count, Los Angeles, CA) method assessed serum insulin levels with monthly quality assurance checks (Diabetes Diagnostic Laboratory, University of Missouri, Columbia, MO). Also, the Hitachi 747-200 (Boehringer Mannheim Diagnostics, Indianapolis, Indiana) with the hexokinase-coupled reaction feature enabled the measurement of fasting glucose levels (Kelley-Hedgepeth et al., 2008).

#### Proinflammatory proteins

A clot-based turbidimetric identification system assessed the fibrinogen level in frozen plasma preserved with citric acid (Medical Laboratory Automation Inc., Mt. Vernon, NY) (Falconi, Gold, & Janssen, 2016). The CRP level was determined by using an ultrasensitive rate immunonephelometry approach with a lower identification limit (0.3 mg/L) (BN100; Dade-Behring, Marburg, Germany).

#### Depression components

Past-week depression components were measured with the Center for Epidemiologic Studies Depression (CES-D) scale (Radloff, 1977). Participants rated items on a 4-point Likert scale (0 = *rarely* to 3 = *most or all of the time*). We focused on three theory-based components derived from a recent factor analytic study in community adults: depressed mood; interpersonal problems; and somatic symptoms (Cosco et al., 2017).

### Statistical analysis

All data analyses were conducted using R version 4.1.0 and RStudio version 1.4.1717 (R Core Team, 2021). Nodes represented components of depression (interpersonal problems, depressed mood, somatic symptoms), proinflammatory proteins and surrogate lipid markers (CRP, fibrinogen, HDL, fasting glucose, insulin, LDL, triglycerides), and covariates [age, estradiol (pg/mL), follicle-stimulating hormone (FSH) (mIU/mL), menopausal status (coded as 1 = premenopausal, 2 = early perimenopausal, 3 = late perimenopausal, 4 = post-menopausal)] (El Khoudary et al., 2016; Persons & Fiedorowicz, 2016). Table 1 shows the descriptive statistics of each node at distinct time-points with the multiply imputed dataset (cf. online Supplementary Table S1 for descriptive statistics with original dataset). Before network estimation, scores for all nodes were rescaled to range from 1 to 4 (matching the CES-D) to minimize biases due to variability differences (Fried et al., 2018). No outliers were identified (i.e. all skewness and kurtosis values were within normal limits).

Next, we used the panel data-graphical vector autoregressive (panelgvar) model (Epskamp, 2020) to determine three networks: (a) within-person temporal (lag-1) network (directed partial associations for the mean within-person effects across time); (b) within-person contemporaneous network (partial associations for the mean within-person effects within a time-point over and above temporal effects); and (c) between-person network (partial associations for stable trait-level differences across time). We fit a non-regularized (unpruned) panelgvar model (Speyer et al., 2022). Model fit was evaluated with these fit statistics: confirmatory fit index (CFI; CFI ⩾ 0.90), Tucker–Lewis index (TLI; TLI ⩾ 0.90), and root mean square error of approximation (RMSEA; RMSEA ⩽ 0.060) (Hu & Bentler, 1999).

As our sample size was large (*n* = 2224), we used an unpruned or non-regularized (*v.* regularized) Gaussian graphical model to interpret network structures because it raises the chances of selecting the true model (Isvoranu & Epskamp, in press). Non-regularized networks were fit using the *qgraph* (Epskamp, Borsboom, & Fried, 2018; Epskamp, Cramer, Waldorp, Schmittmann, & Borsboom, 2012) and *psychonetrics* (Epskamp, 2020) R packages. We uploaded analytic data syntax to OSF (https://osf.io/upkyr/). The non-regularized graphical least absolute shrinkage and selection operator (graphical LASSO) was used to estimate the structure of 100 regularized network models from sparse to dense (Epskamp, Kruis, & Marsman, 2017; Moriarity et al., 2021a; Williams & Rast, 2020).

To determine the accuracy of network edges, we computed the 95% confidence intervals (CIs) of the edge weights with 1000 bootstrap samples (Costenbader & Valente, 2003). Furthermore, only statistically significant edges (*p* < 0.001) and edges included ⩾50% of the time across 1000 bootstrap samples were regarded as stable (Betz et al., 2020; Epskamp, 2020). Cohen's *d* effect sizes were calculated to ease interpretation (Dunlap, Cortina, Vaslow, & Burke, 1996; Rosenthal, 1994). Based on the literature (Mac Giollabhui et al., 2021), *d* ⩾ 0.100 was interpreted as meaningful. We rendered edges that were accurate, stable, and with *d* ⩾ 0.100 as significant. In addition, the Fruchterman–Reingold algorithm was used to organize the networks by locating the largest associations in the center and weaker associations toward the boundary and placing nodes with stronger relations closer to each other (Fruchterman & Reingold, 1991). Line thickness indicates the strength of association. Although bold blue lines signal positive relations, red dotted lines reflect negative ones. To test H1 formally, we used robust variance estimation (RVE) (Tanner-Smith, Tipton, & Polanin, 2016) to determine if substantial effect sizes consistent with vulnerability (*v.* scar) theories were statistically significantly different. To evaluate H2, we utilized RVE to test the existence of significant effect size differences between substantial edges that included somatic symptoms, proinflammatory proteins, and their interaction.^*^^1^

## Results

### CLPN model fit evaluation

The non-regularized CLPN model had good fit (CFI = 1.00, TLI = 0.95, RMSEA = 0.000, 90% [CI] [0.000–0.000]).

### Accuracy and stability of networks

Online Supplementary Figs S2(a)–S2(c) present the 95% CI plot that indicates the accuracy of all edges for the within-person temporal network, within-person contemporaneous network, and between-person network, respectively. The percentages of 95% CI for edges that did not cross the 0 value were 95.8% (182/190) for the temporal network, 98.9% (90/91) for the contemporaneous network, and 96.7% (88/91) for the between-person network. Online Supplementary Tables S2(a)–S2(c) show the partial correlation statistics of each network, and online Supplementary Tables S3(a)–S3(c) show the frequency that each edge was included across all 1000 bootstrap samples. The frequency of edges included in ⩾50% of all bootstrap samples was 129 out of 196 edges (65.8%) for the temporal network, 54 out of 91 edges (59.3%) for the contemporaneous network, and 60 out of 91 edges (65.9%) for the between-person network. Thus, all networks showed a good degree of accuracy and stability.

### Within-person temporal (lag-1) network

**Fig. 1.**
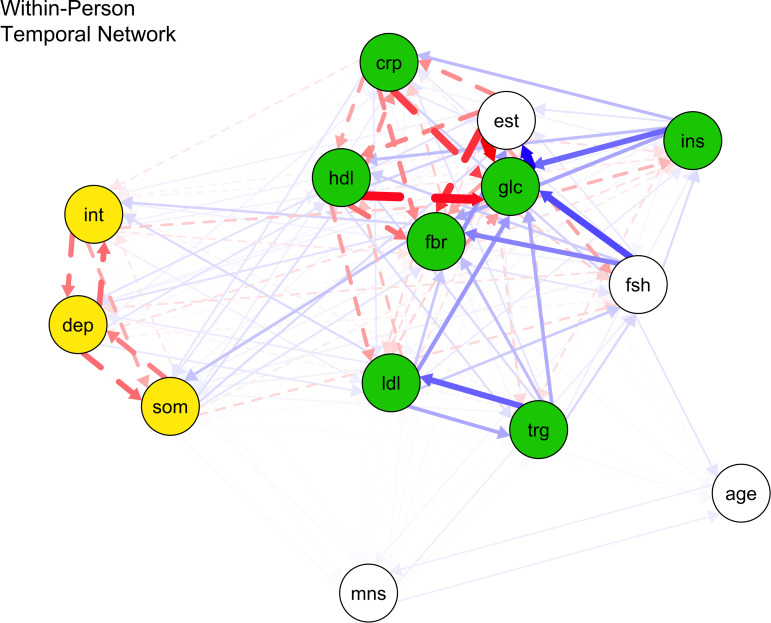
Within-person temporal network of proinflammatory proteins, lipid markers, and depression nodes. *Note*: crp, C-reactive protein; dep, depressed mood; fbr, fibrinogen; glc, fasting glucose; hdl, high density lipoprotein; ins, insulin; int, interpersonal problems; lip, lipid marker composite; ldl, low density lipoprotein; som, somatic symptoms; trg, triglycerides. Blue bold lines indicate statistically significant positive relations, whereas red dotted lines signal statistically significant negative relations and line boldness and thickness reflect strength of associations.

Table 2 shows the parameter estimates for the within-person temporal (lag-1) network edges across distinct depression and surrogate immunometabolism constructs. Figure 1 displays all estimated fixed-effect within-person network standardized partial correlations. Online Supplementary Table S4 displays parameter estimates of all lag-1 directed network edges within and across constructs.

**Table 2.** Cross-construct cross-lagged directed edges of within-person temporal (lag-1) network

Node-out	Node-in	Edge	*p*	*d*	Node-out	Node-in	Edge	*p*	*d*
dep	fbr	0.00197	0.000	0.081	fbr	dep	−0.00054	0.000	−0.076
dep	crp	0.00012	0.000	0.004	**fbr**	**som**	**0.00166**	**0.000**	**0.156**
dep	glc	0.00075	0.000	0.029	fbr	int	−0.00172	0.000	−0.365
dep	ins	0.00090	0.000	0.008	**crp**	**dep**	**0.00081**	**0.000**	**1.072**
dep	trg	−0.00091	0.000	−0.009	**crp**	**som**	**0.00110**	**0.000**	**1.812**
dep	ldl	0.00131	0.000	0.094	**crp**	**int**	**0.00033**	**0.000**	**2.112**
dep	hdl	−0.00031	0.000	−0.008	glc	dep	−0.00201	0.000	−0.289
**som**	**fbr**	**0.00388**	**0.000**	**0.188**	glc	som	−0.00006	0.000	−0.005
som	crp	0.00164	0.000	0.056	glc	int	−0.00345	0.000	−0.715
som	glc	0.00061	0.000	0.025	ins	dep	−0.00009	0.000	−0.245
som	ins	0.00074	0.000	0.009	ins	som	−0.00135	0.000	−6.245
som	trg	0.00064	0.000	0.006	ins	int	−0.00033	0.000	−0.708
som	ldl	0.00087	0.000	0.099	trg	dep	0.00000	0.000	−0.004
som	hdl	0.00157	0.000	0.046	**trg**	**som**	**0.00014**	**0.000**	**0.131**
**int**	**fbr**	**0.00284**	**0.000**	**0.129**	trg	int	0.00007	0.000	0.071
int	crp	−0.00159	0.000	−0.058	**ldl**	**dep**	**0.00155**	**0.000**	**0.251**
int	glc	−0.00086	0.000	−0.036	ldl	som	−0.00043	0.000	−0.074
int	ins	−0.00096	0.000	−0.006	**ldl**	**int**	**0.00101**	**0.000**	**0.327**
int	trg	−0.00105	0.000	−0.011	**hdl**	**dep**	**0.00172**	**0.000**	**0.196**
**int**	**ldl**	**0.00236**	**0.000**	**0.331**	**hdl**	**som**	**0.00214**	**0.000**	**0.162**
int	hdl	0.00009	0.000	0.003	**hdl**	**int**	**0.00082**	**0.000**	**0.134**

*Note*: crp, C-reactive protein; dep, depressed mood; fbr, fibrinogen; glc, fasting glucose; hdl, high density lipoprotein; ins, insulin; int, interpersonal problems; lip, lipid marker composite; ldl, low density lipoprotein; som, somatic symptoms; trg, triglycerides. Bold values reflect statistically significant cross-construct edges (i.e., *p* < 0.001, edges were included ⩾50% of the time across 1000 bootstrap samples and *d* ⩾ 0.100).

#### Scar theories

Within persons, depressed mood did not stably predict other immunometabolism markers at the next time-point. However, within-person increased somatic symptoms significantly predicted future increased fibrinogen (*d* = 0.188) (*p* < 0.001) rather than other lipid markers and proinflammatory proteins. Also, heightened interpersonal problems significantly predicted future higher fibrinogen (*d* = 0.129) and LDL (*d* = 0.331) (all *p*s < 0.001) instead of other lipid markers and proinflammatory proteins.

#### Vulnerability models

These two surrogate immunometabolism markers significantly predicted all future depression nodes: (a) CRP (higher CRP → greater depressed mood: *d* = 1.072; higher CRP → greater somatic symptoms: *d* = 1.812; higher CRP → greater interpersonal problems: *d* = 2.112) (all *p*s < 0.001); and (b) HDL (higher HDL → stronger depressed mood: *d* = 0.196; higher HDL → stronger somatic symptoms: *d* = 0.162; higher HDL → stronger interpersonal problems: *d* = 0.134) (all *p*s < 0.001). Also, higher depressed mood was significantly predicted by previous higher LDL levels (*d* = 0.251, *p* < 0.001) instead of fibrinogen, glucose, insulin, and triglycerides. Greater somatic symptoms were significantly predicted by prior higher levels of fibrinogen (*d* = 0.156) and triglycerides (*d* = 0.174) (all *p*s < 0.001), but not fasting glucose, insulin, and LDL. More interpersonal problems were significantly predicted by prior higher LDL (*d* = 0.436, *p* < 0.001), but not fibrinogen, insulin, fasting glucose, and triglycerides.

The effect sizes from scar and vulnerability models did not significantly differ from one another (*β* = 0.081, 95% CI −0.207 to 0.368). Thus, the findings did not support H1.

### Within-person contemporaneous network

**Fig. 2.**
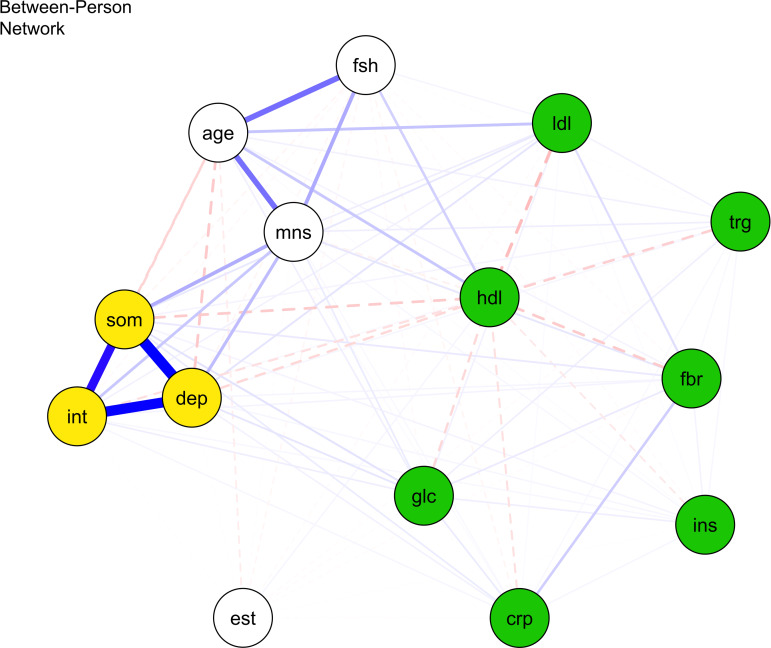
Within-person contemporaneous network of proinflammatory proteins, lipid markers, and depression nodes. *Note*: crp, C-reactive protein; dep, depressed mood; fbr, fibrinogen; glc, fasting glucose; hdl, high density lipoprotein; ins, insulin; int, interpersonal problems; lip, lipid marker composite; ldl, low density lipoprotein; som, somatic symptoms; trg, triglycerides. Blue bold lines indicate statistically significant positive relations, whereas red dotted lines signal statistically significant negative relations and line boldness and thickness reflect strength of associations.

Figure 2 and online Supplementary Table S5 show all contemporaneous network edges parameter estimates and statistics after adjusting for within-person temporal relations and between-person differences. Within persons, greater depressed mood was significantly related to higher fasting glucose (*d* = 0.298, *p* < 0.001), but not CRP, fibrinogen, insulin, triglycerides, HDL, and LDL. Also, greater somatic symptoms were significantly associated with higher fasting glucose (*d* = 3.586, *p* < 0.001), but not fibrinogen, CRP, insulin, triglycerides, LDL, and HDL. Additionally, within-person greater interpersonal problems were significantly correlated with higher fibrinogen (*d* = 1.029), fasting glucose (*d* = 1.055), and HDL (*d* = 0.181) (all *p*s < 0.001), but not CRP, insulin, triglycerides, and LDL levels.

### Between-person network

**Fig. 3.**
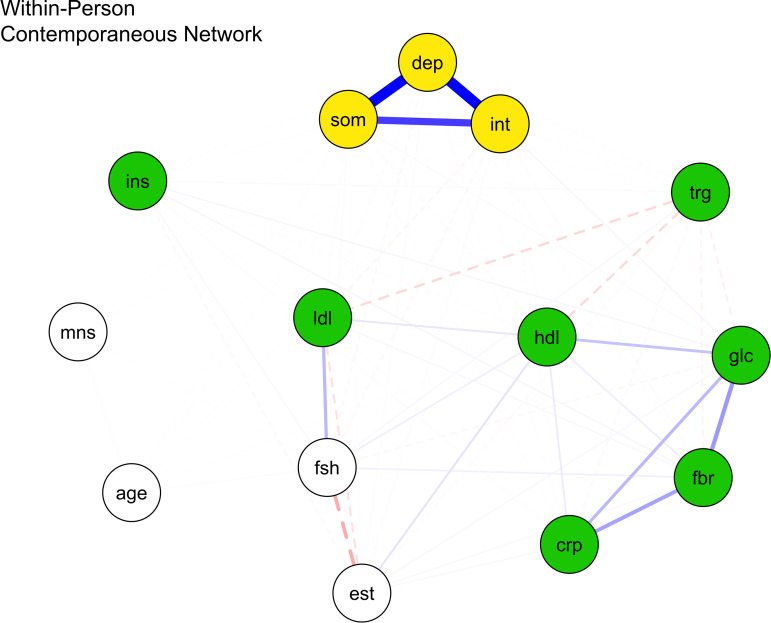
Between-person network of proinflammatory proteins, lipid markers, and depression nodes. *Note*: crp, C-reactive protein; dep, depressed mood; fbr, fibrinogen; glc, fasting glucose; hdl, high density lipoprotein; ins, insulin; int, interpersonal problems; lip, lipid marker composite; ldl, low density lipoprotein; som, somatic symptoms; trg, triglycerides. Blue bold lines indicate statistically significant positive relations, whereas red dotted lines signal statistically significant negative relations and line boldness and thickness reflect strength of associations.

Figure 3 and online Supplementary Table S6 show that between persons, stronger depressed mood was significantly related to higher CRP (*d* = 0.205) and fasting glucose (*d* = 0.138) (all *p*s < 0.001), but not fibrinogen, triglycerides, insulin, LDL, and HDL levels. Between persons, stronger somatic symptoms were significantly associated with higher CRP (*d* = 0.240), fasting glucose (*d* = 0.447), insulin (*d* = 0.231) (all *p*s < 0.001), but not fibrinogen, triglycerides, HDL, and LDL. Also, interpersonal problems were not stably related to any immunometabolism markers between persons. Inconsistent with H2, the strength of associations did not differ between significant edges with somatic symptoms (*v.* depressed mood and interpersonal problems) (*β* = −0.003, 95% CI −0.130 to 0.125), proinflammatory proteins (*v.* proxy lipids) (*β* = 0.0144, 95% CI −0.128 to 0.157), and their interaction (*β* = 0.007, 95% CI −0.261 to 0.274).

## Discussion

Contrary to our hypotheses, findings provided consistent evidence for vulnerability models and scar theories, with small-to-large effect sizes. Furthermore, somatic symptoms, depressed mood, and interpersonal problems had similarly strong positive relations with proinflammatory proteins and proxy lipid markers. We offer potential theoretical accounts on this topic based on outcomes produced by the current study's largely data-driven, cutting-edge CLPN. The within-person temporal network, rather than within-person contemporaneous and between-person networks, takes precedence when interpreting results because it provides directionality information.

Some notable temporal network relations emerged between components of depression and proinflammatory proteins. First, replicating and extending a recent meta-analysis with similar findings (Mac Giollabhui et al., 2021), higher CRP unidirectionally predicted later increased depressed mood, somatic symptoms, and interpersonal problems, but not vice versa. We observed large effect sizes of CRP (*v.* other proxy immunometabolism markers) predicting depression components in the within-person temporal networks (*d* = 1.072–2.112). The unique biological properties of CRP (e.g. cardiovascular risk-enhancing attributes, increased fat storage) might contribute to those large effects, as evidenced by Mendelian randomization genetic (e.g. Khandaker et al., 2020) and related studies (Castanon, Lasselin, & Capuron, 2014) with hundreds of thousands of participants. Prognostically, suboptimal CRP and associated markers (e.g. fibrinogen, HDL, triglycerides, LDL levels) are probably proinflammatory proteins and surrogate lipid markers driving the etiology of depression. Thus, it is possible that modifying these proxy immunometabolism markers might efficiently treat depression and improve immunometabolism profiles. Also, within persons, contemporaneous networks revealed large positive cross-sectional effect sizes between somatic symptoms and glucose as well as interpersonal problems and glucose and fibrinogen (*d* = 1.029–3.589) above and beyond temporal effects. Such outliers suggest that the distinctive depression-associated mechanisms of excessive fibrinogen (e.g. increased arterial plaques and clots) and glucose (e.g. metabolism-altering characteristics) merit attention (Kucukgoncu et al., 2019; Von Känel, Bellingrath, & Kudielka, 2009).

Another notable observation was that there were larger effect sizes at the within- (*v.* between-) person level (i.e. average significant *d* = 0.731 *v.* 0.252). Such findings suggest that biological psychiatry can profit from conducting more studies with within-subject designs that capture person-specific fluctuations since effect size magnitudes can vary at the individual difference and within-person levels (Renna et al., 2020). Although longitudinal between-person analyses allow an inference that immunometabolism at a time-point predicts later depression across a sample, such group-level patterns might not extend to individuals across time (Wright & Woods, 2020).

Additionally, fibrinogen had a positive and small reciprocal effect on somatic symptoms over time. Such a result extends evidence for fibrinogen levels positively predicting depression indices (e.g. major depressive disorder) (Zainal & Newman, 2021b). Our findings support the idea that inflammatory processes are more pronounced in atypical (*v.* melancholic/mood-focused) depression characterized by bodily symptoms (Penninx, 2017). They also buttress the ‘sickness behavior’ hypothesis that somatic symptoms (e.g. psychomotor slowing, restless sleep) substantially predict increased proinflammatory proteins (Iob, Kirschbaum, & Steptoe, 2020).

Overall, our results highlight the importance of clarifying *unique* depression components that specific proinflammatory proteins positively impact. Plausibly, increased CRP and fibrinogen predicted heightened depression components, particularly somatic symptoms, by producing more proinflammatory cytokines from peripheral blood mononuclear cells (e.g. IL-6, tumor necrosis factor-*α*) (Haroon, Raison, & Miller, 2012). Proinflammatory cytokines might trigger and increase the enzyme indoleamine-2,3-dioxygenase, which depletes monoamine precursors (i.e. antecedents of serotonin and dopamine such as tryptophan) by breaking it down into kynurenine (Felger, 2018). Eventually, reduced serotonin, dopamine, and norepinephrine synthesis and modified apoptosis and oxidative stress (Lamers et al., 2020) could contribute to elevated depression. Future basic science research should evaluate these notions.

Notably, temporal networks showed that excessive HDL predicted all depression components measured herein but not conversely. Furthermore, temporal networks revealed positive feedback loops between LDL and depressed mood and LDL and interpersonal problems, but not LDL and somatic symptoms. Also, elevated triglycerides preceded more somatic symptoms (*v.* other depression nodes) than vice versa, suggesting that this is an event that could occur in both community-dwelling adult women and men (Xu et al., 2021). Such observations agree that reducing hypertriglyceridemia is essential to treat and prevent the onset or recurrence of physical aspects of depression (Hamer, Batty, & Kivimaki, 2012). The state-of-the-art network analysis thus offers much information on the direction, magnitude, and possible reciprocal influence(s) among components of depression and surrogate lipid markers. Our results expand on cross-sectional meta-analytic evidence that HDL positively correlated with depression only among women (Shin, Suls, & Martin, 2008) and network analytic evidence that heightened HDL (*v.* LDL and total cholesterol) coincided with more depressed mood (Jia et al., 2020). They also add to accruing evidence for the role of proxy markers of metabolic syndrome and poor glycemic control serving as risk factors for elevated depression in community adults (Mezuk, Eaton, Albrecht, & Golden, 2008; Watson et al., 2021).

Suboptimal levels of unique lipid markers heightened the risk of experiencing more distinct aspects of depression later, likely by dysregulating the HPA via excessive or blunted (*v.* optimal) cortisol production (Mansur, Brietzke, & McIntyre, 2015). Other tenable mechanisms include decreased neurogenesis in reward- and executive functioning-related brain regions and connectivity between physiological states and synaptic plasticity (Goldsmith et al., 2020; Hamer et al., 2019; Zainal & Newman, in press). Plausibly, these processes can unfold with and without chronic social stressors and relate to somatic aspects (e.g. appetite changes, fatigue) of depression that often co-occur with motivational deficits and social withdrawal (Coccurello, 2019). Future prospective network analyses should examine these ideas.

Partially consistent with scar theories, somatic symptoms and interpersonal issues, but not depressed mood, preceded higher fibrinogen levels. More interpersonal problems, but not depressed mood and somatic symptoms, also forecasted increased LDL. Results extend evidence that more daily positive interpersonal events dovetailed with future reduced CRP and fibrinogen among women but not men (Sin, Graham-Engeland, & Almeida, 2015). They also build on evidence that rises in HDL or LDL levels (indicators of the buildup of fatty plaques in heart arteries) predicted depression in community adult women instead of men (Beydoun et al., 2015) and more cardiovascular events and rapid cognitive decline (Hua, Ma, Li, Zhong, & Xie, 2021). Most importantly, findings suggest improving lifestyle patterns to lessen depression and prevent dyslipidemia and heightened inflammation.

Study limitations merit attention. First, the all-female sample precluded the generalization of findings to the general population. Future studies should examine how sex assigned at birth might influence our CLPN-derived results due to documented sex differences in proinflammatory proteins, lipid markers, problem- *v.* emotion-focused coping approaches, and their interactions (Shimanoe et al., 2018). For example, sex could moderate within-person CRP-depression associations (Das, 2020) and relate to the hypothalamic–pituitary–gonadal axis, corticotropic-releasing hormone, cell death programming, and mitochondrial differences (Dantzer et al., 2021). Although women usually consume fatty acids in most cell metabolism processes, men mainly use amino acids and proteins (Demarest & McCarthy, 2015). For these and related biopsychosocial reasons, heightened depression occurs in more women than men (Shimamoto & Rappeneau, 2017), necessitating the recruitment of both genders in future studies. Second, as the current study was a secondary analysis, we were limited to available data. Other related chronic low-grade systemic proinflammatory proteins (e.g. IL-6), endocrine markers, and psychopathology components might have contributed to the current pattern of results. For example, IL-6 is instrumental in CRP and fibrinogen production, and inhibiting IL-6 with monoclonal antibodies affects lipid markers (Raison, Knight, & Pariante, 2018). Also, although controlling for age did not affect the results in this middle-aged sample, network associations might be more potent in middle-aged compared to younger adult women (Walker et al., 2021). Nonetheless, study strengths include the large sample size and the cutting-edge CLPN that separated within- and between-person relations and offered more information than traditional statistics. Moreover, our analyses adjusted for age, estradiol, FSH, and menopausal status.

Cognitive-behavioral therapies (CBTs) that raise the consumption of foods with high soluble dietary fiber (e.g. oat bran, rye bran), reduce intake of sugary or low-fiber foods, and promote regular physical activity may facilitate those aims (Johansson-Persson et al., 2014; Li et al., 2017). Also, clinical science can profit from testing the efficacy of encouraging the consumption of a Mediterranean diet (e.g. olive oil, fish, fruits, vegetable) (Abenavoli et al., 2018) and improving sleep using evidence-based CBT strategies (Irwin et al., 2014). Furthermore, findings highlight how optimizing immunometabolism profiles require enhancing social support (e.g. reducing loneliness), social engagement, and related contextual variables (cf. interpersonal theories; Walker, Ploubidis, and Fancourt, 2019; Wiebe, Helgeson, and Berg, 2016). Mounting evidence indicates that these CBT approaches could alleviate depression and enhance immunometabolism profiles long-term (Shomaker et al., 2017), which merit more attention.

## Footnotes

* The notes appear after the main text.

## Note

1. Due to space constraints, we offer more details on the procedures, measures, and statistical analyses in Appendix A of the online Supplementary material.

## Supplementary material

For supplementary material accompanying this paper visit https://doi.org/10.1017/S003329172200232X. click here to view supplementary material
