# Supplementary material for: Prospective network analysis of proinflammatory proteins, lipid markers, and depression components in midlife community women
Source: Psychol Med. 2022 Aug 4;53(11):5267–78. doi: 10.1017/S003329172200232X (PMC9898473; doi:10.1017/S003329172200232X)
Supplement: Supplementary file 1 [file S003329172200232Xsup.zip › S003329172200232Xsup002.docx]

# Supplementary Materials

# Appendix A

## Participants

The proportion of participants ethnically identified as Caucasian, African American, Hispanic, Chinese, and Japanese were 47%, 28%, 8%, 8%, and 8%, respectively. In addition, whereas 3% reported they did not know or declined to state their past annual total household income, 47% disclosed earning $49,999 or below, and 50% $50,000 or more.

## Procedures

Blood specimens were collected via blood draws following a 12-hour fast. The blood draw took place on the morning after fasting to standardize the protocol. For menstruating women, specimens were collected between days 2 and 5 of the menstrual cycle (i.e., the early follicular phase) to reduce hormonal variations. Subsequently, specimens were shipped with dry ice at -20°C to project-affiliated laboratories, where the plasma was frozen at -80°C (Medical Research Laboratories, Highland Heights, Kentucky) for later processing in systematic batches (Myers et al., 1989). Storage time of specimens in the freezer and their processing time took place within three months and did not differ substantially between and within participants and timepoints (El Khoudary et al., 2016a).

## Measures

**Proinflammatory proteins.** The within-run coefficients of CRP were 10-12% and 5-7%, at CRP amounts of 0.5 and 22.0mg/L, respectively (Kelley-Hedgepeth et al., 2008).

**Depression components.** Each node indicated a mean composite score of items averaged for these components: depressed mood (Cronbach’s α = 0.937–0.943); interpersonal problems (α = 0.711–0.739); and somatic symptoms (α = 0.849–0.860). Its scores have shown excellent retest reliability and convergent and discriminant validity (Cosco, Prina, Stubbs, & Wu, 2017).

## Statistical Analysis

As part of the preprocessing, missing data (comprising 11.13% of total observations) was managed with multiple imputation using the *mice R* package (van Buuren & Groothuis-Oudshoorn, 2011); the gold standard given the total amount of missingness and with the high likelihood the current data were missing at random (Lee & Shi, 2021). Data were aggregated across 100 imputed datasets, each with a maximum of 10 iterations.

Community structure detection analysis with the Walktrap algorithm (Pons & Latapy, 2005) revealed three communities of nodes (refer to Figure S1).

We selected our covariates consistent based on evidence that they could affect proinflammatory proteins and surrogate lipid markers levels over time (El Khoudary et al., 2016b; Persons et al., 2016).

The panelgvar-model computes directed and undirected network edges, above and beyond trait-level between-person and autoregressive effects and baseline scores by adding a random intercept (Hamaker, Kuiper, & Grasman, 2015; Wild et al., 2010). A better understanding of within-person effects is important as they can uncover treatment mechanisms explaining relations among depression components and identify chief treatment targets. However, the panelgvar model can only offer average population-level (fixed effect) insights instead of individual-level, within-person temporal dynamics.

The non-regularized graphical LASSO approach iteratively fits a non-regularized network for each model using graphical LASSO without regularization, but with zeros constrained based on the network structure. To acquire unbiased parameter estimates, we used maximum likelihood estimation. Further, the non-regularized (vs. the more popular regularized) graphical LASSO network estimation approach has higher specificity (lower false positives) to empirically identify unique non-zero edges likely derived from the true population (Williams & Rast, 2020). It does not assume that the true network is sparse (Epskamp et al., 2017). Moreover, non-regularized (vs. regularized) network models produce fewer biases in parameter estimates (e.g., dropping of edges with proinflammatory proteins) (Moriarity et al., 2021a).

Cohen’s *d* effect sizes were calculated to ease interpretation using the formulas: (a) within-person Cohen’s *d* = (β/*SD*(β))*√(2/*n*) (Dunlap et al., 1996); (b) between-person *d* = (2*(β/*SD*(β)))/√(*df*) (Rosenthal, 1994). β is the beta weight regression, *SD* is the standard deviation of the beta weight, *n* is the total number of observations, and *df* is the degrees of freedom of the error term.

Table S1(a)

*Descriptive Statistics of Network Nodes Across All Waves for Original Dataset (Non-Imputed Data)*

|  | Wave 1 | | | Wave 2 | | | Wave 4 | | | Wave 6 | | | Wave 8 | | |
| --- | --- | --- | --- | --- | --- | --- | --- | --- | --- | --- | --- | --- | --- | --- | --- |
|  | *n* | *M/%* | (SD) | *n* | *M/%* | (SD) | *n* | *M/%* | (SD) | *n* | *M/%* | (SD) | *n* | *M/%* | (SD) |
| Age | 2224 | 45.964 | (2.674) | 2095 | 47.036 | (2.687) | 2081 | 49.049 | (2.682) | 2100 | 51.073 | (2.688) | 2224 | 53.094 | (2.689) |
| Depressed mood | 2224 | 1.524 | (0.627) | 2089 | 1.471 | (0.598) | 2079 | 1.454 | (0.586) | 2092 | 1.461 | (0.588) | 2092 | 1.426 | (0.586) |
| Somatic symptoms | 2089 | 1.778 | (0.575) | 2089 | 1.767 | (0.619) | 2079 | 1.749 | (0.611) | 1963 | 1.794 | (0.642) | 2092 | 1.692 | (0.584) |
| Interpersonal problems | 2224 | 1.426 | (0.621) | 2089 | 1.372 | (0.578) | 2079 | 1.378 | (0.579) | 2092 | 1.356 | (0.569) | 2092 | 1.349 | (0.579) |
| Fibrinogen | 2162 | 1.920 | (0.322) | 1946 | 1.917 | (0.334) | 1694 | 2.241 | (0.249) | 1740 | 2.062 | (0.298) | 835 | 1.997 | (0.387) |
| CRP | 2184 | 1.104 | (0.170) | 1993 | 1.077 | (0.149) | 1818 | 1.306 | (0.186) | 1821 | 1.110 | (0.174) | 1843 | 1.193 | (0.307) |
| Glucose | 2122 | 1.350 | (0.227) | 1945 | 1.282 | (0.249) | 1803 | 1.831 | (0.225) | 1779 | 1.524 | (0.248) | 1814 | 1.296 | (0.200) |
| Insulin | 2116 | 1.067 | (0.097) | 1925 | 1.066 | (0.106) | 1799 | 1.085 | (0.083) | 1664 | 1.133 | (0.168) | 1718 | 1.092 | (0.139) |
| Triglycerides | 2115 | 1.203 | (0.196) | 1941 | 1.188 | (0.171) | 1809 | 1.139 | (0.152) | 1774 | 1.199 | (0.183) | 1793 | 1.239 | (0.170) |
| LDL | 2097 | 2.140 | (0.391) | 1922 | 2.168 | (0.408) | 1791 | 2.170 | (0.433) | 1751 | 2.214 | (0.406) | 1772 | 2.312 | (0.468) |
| HDL | 2209 | 2.056 | (0.458) | 2032 | 2.032 | (0.460) | 1877 | 2.256 | (0.421) | 1839 | 2.021 | (0.396) | 1845 | 2.091 | (0.496) |
| FSH | 2220 | 1.257 | (0.281) | 2030 | 1.330 | (0.371) | 1876 | 1.344 | (0.346) | 1817 | 1.445 | (0.370) | 1895 | 1.540 | (0.345) |
| Menopausal status | |  |  |  |  |  |  |  |  |  |  |  |  |  |  |
| Pre | 1236 | 55.576 | – | 829 | 37.275 | – | 643 | 28.912 | – | 409 | 18.390 | – | 50 | 2.248 | – |
| Early Peri | 988 | 44.424 | – | 1254 | 56.385 | – | 1078 | 48.471 | – | 805 | 36.196 | – | 576 | 25.899 | – |
| Late Peri | – | – | – | 99 | 4.451 | – | 199 | 8.948 | – | 234 | 10.522 | – | 241 | 10.836 | – |
| Post | – | – | – | 42 | 1.888 | – | 304 | 13.669 | – | 776 | 34.892 | – | 1357 | 61.016 | – |
| Estradiol | 2220 | 1.144 | (0.168) | 2030 | 1.236 | (0.284) | 1872 | 1.217 | (0.270) | 1770 | 1.041 | (0.093) | 1888 | 1.090 | (0.176) |
|  |  |  |  |  |  |  |  |  |  |  |  |  |  |  |  |

*Note.* *M* = mean; *SD* = standard deviation; LDL = low density lipoprotein; HDL = high density lipoprotein; CRP = C-reactive protein; FSH = follicle-stimulating hormone. All scores have been rescaled to range from 1 to 4.

Table S1(b)

*Descriptive Statistics of Other Relevant Variables Not Included into Final Analyses*

|  | Wave 1 | | | Wave 2 | | | Wave 4 | | | Wave 6 | | | Wave 8 | | |
| --- | --- | --- | --- | --- | --- | --- | --- | --- | --- | --- | --- | --- | --- | --- | --- |
|  | *n* | *M/%* | (SD) | *n* | *M/%* | (SD) | *n* | *M/%* | (SD) | *n* | *M/%* | (SD) | *n* | *M/%* | (SD) |
| BMI | 2198 | 27.929 | (7.273) | 2050 | 27.97 | (7.157) | 1992 | 28.515 | (7.319) | 1916 | 28.816 | (7.377) | 1949 | 28.855 | (7.329) |
| Thyroid | 137 | 6.160 | – | 134 | 6.030 | – | 181 | 8.138 | – | 176 | 7.914 | – | 217 | 9.757 | – |
| Diabetes | 73 | 3.282 | – | 92 | 4.137 | – | 92 | 4.137 | – | 145 | 6.520 | – | 205 | 9.218 | – |
| High cholesterol | 404 | 18.165 | – | 221 | 9.937 | – | 247 | 11.106 | – | 314 | 14.119 | – | 463 | 20.818 | – |
| HBP | 293 | 13.174 | – | 319 | 14.344 | – | 405 | 18.210 | – | 459 | 20.638 | – | 571 | 25.674 | – |
|  |  |  |  |  |  |  |  |  |  |  |  |  |  |  |  |

*Note.* BMI = body mass index; HBP = high blood pressure; *M* = mean; *SD* = standard deviation.

Table S2

*Correlation Statistics of Panel GVAR Networks*

1. Within-Person Temporal Networks

|  | dep | som | int | fbr | crp | glc | ins | trg | ldl | hdl | age | fsh | mns | est |
| --- | --- | --- | --- | --- | --- | --- | --- | --- | --- | --- | --- | --- | --- | --- |
| dep | 0.08579 | -0.00877 | -0.00900 | 0.00335 | 0.00034 | 0.00115 | 0.00512 | -0.00379 | 0.00180 | -0.00066 | -0.00296 | -0.00351 | 0.01578 | 0.00395 |
| som | -0.01060 | 0.08132 | -0.00562 | 0.00690 | 0.00485 | 0.00092 | 0.00422 | 0.00276 | 0.00120 | 0.00320 | -0.00026 | 0.00152 | 0.01429 | 0.00141 |
| int | -0.01096 | -0.00156 | 0.07803 | 0.00534 | -0.00488 | -0.00157 | -0.00587 | -0.00457 | 0.00338 | 0.00023 | 0.00046 | 0.00152 | 0.01512 | 0.00204 |
| fbr | -0.00028 | 0.00090 | -0.00091 | 0.10171 | -0.01381 | -0.00617 | 0.01906 | 0.01042 | 0.00286 | -0.01155 | -0.00568 | 0.00787 | 0.00764 | -0.01800 |
| crp | 0.00028 | 0.00037 | 0.00010 | -0.00202 | 0.10013 | -0.00076 | 0.00906 | 0.00241 | 0.00033 | -0.00530 | -0.01500 | 0.00080 | -0.00055 | -0.00572 |
| glc | -0.00113 | -0.00004 | -0.00189 | -0.00683 | -0.02390 | 0.09571 | 0.03448 | 0.01378 | 0.00569 | -0.02166 | 0.01527 | 0.01230 | 0.00310 | -0.02611 |
| ins | -0.00002 | -0.00025 | -0.00006 | -0.00069 | 0.00046 | -0.00162 | 0.11032 | 0.00081 | 0.00034 | -0.00062 | -0.00415 | 0.00073 | 0.01190 | -0.00083 |
| trg | -0.00001 | 0.00003 | 0.00001 | -0.00017 | 0.00043 | -0.00108 | -0.00299 | 0.10435 | 0.00194 | 0.00109 | -0.01387 | 0.00009 | 0.00846 | 0.00045 |
| ldl | 0.00110 | -0.00032 | 0.00066 | -0.00346 | 0.00293 | -0.00360 | 0.00187 | 0.03399 | 0.09448 | -0.00983 | -0.00363 | -0.00017 | 0.01310 | -0.00323 |
| hdl | 0.00083 | 0.00102 | 0.00037 | 0.00047 | -0.00901 | 0.00020 | 0.01205 | 0.00482 | -0.00299 | 0.10003 | -0.00401 | 0.00269 | 0.00163 | -0.00868 |
| age | -0.00200 | -0.00200 | -0.00200 | 0.00200 | 0.00200 | 0.00200 | 0.00200 | 0.00200 | 0.00200 | 0.00200 | 0.12153 | 0.00200 | 0.05803 | -0.00200 |
| fsh | -0.00052 | -0.00160 | 0.00017 | 0.00197 | 0.00533 | -0.00316 | 0.00188 | 0.00678 | 0.00297 | 0.00030 | 0.02389 | 0.11448 | 0.04931 | -0.00800 |
| mns | 0.00001 | 0.00000 | 0.00000 | 0.00000 | 0.00000 | 0.00000 | 0.00001 | 0.00000 | 0.00000 | 0.00000 | 0.94064 | 0.00002 | 0.12076 | -0.00001 |
| est | 0.00027 | 0.00112 | 0.00042 | 0.00631 | 0.00215 | 0.01508 | 0.00468 | -0.00586 | -0.00123 | 0.00296 | 0.01289 | 0.00113 | 0.01531 | 0.09713 |
|  |  |  |  |  |  |  |  |  |  |  |  |  |  |  |

*Note.* age = age at unique time-point; crp = C-reactive protein; dep = depressed mood; est = estradiol; fsh = follicle-stimulating hormones; fbr = fibrinogen; glc = fasting glucose; GVAR = graphical vector autoregressive models; hdl = high density lipoprotein; ins = insulin; int = interpersonal problems; lip = lipid marker composite; ldl = low density lipoprotein; mns = menopausal status; som = somatic symptoms; trg = triglycerides. This table shows estimates of partial directed associations. Diagonal numbers indicate autocorrelations. Lower triangle indicates estimates of variables in the leftmost column predicting future lag-1 variables in the topmost row. Upper triangle indicates the opposite temporal (lag-1) relations.

Table S2 (*continued*)

*Correlation Statistics of Panel GVAR Networks*

1. Within-Person Contemporaneous Networks

|  | dep | som | int | fbr | crp | glc | ins | trg | ldl | hdl | age | fsh | mns | est |
| --- | --- | --- | --- | --- | --- | --- | --- | --- | --- | --- | --- | --- | --- | --- |
| dep | 0.00000 | 0.11761 | 0.11736 | -0.00162 | -0.00158 | 0.00077 | -0.00085 | -0.00152 | -0.00242 | 0.00041 | -0.00042 | -0.00335 | 0.00051 | 0.00085 |
| som | 0.11761 | 0.00000 | 0.07634 | -0.00057 | -0.00148 | 0.00199 | -0.00042 | -0.00115 | -0.00141 | -0.00238 | -0.00034 | 0.00074 | 0.00064 | 0.00021 |
| int | 0.11736 | 0.07634 | 0.00000 | 0.00084 | -0.00100 | 0.00301 | -0.00005 | -0.00158 | -0.00390 | 0.00029 | -0.00035 | -0.00258 | 0.00043 | 0.00090 |
| fbr | -0.00162 | -0.00057 | 0.00084 | 0.00000 | 0.03418 | 0.03811 | 0.00356 | -0.00531 | 0.00277 | 0.00646 | 0.00008 | 0.00543 | 0.00017 | 0.00212 |
| crp | -0.00158 | -0.00148 | -0.00100 | 0.03418 | 0.00000 | 0.02533 | 0.00079 | -0.00263 | -0.00049 | 0.00749 | 0.00004 | -0.00003 | 0.00005 | 0.00208 |
| glc | 0.00077 | 0.00199 | 0.00301 | 0.03811 | 0.02533 | 0.00000 | 0.00368 | -0.00607 | -0.00318 | 0.02154 | 0.00012 | -0.00354 | 0.00014 | 0.00275 |
| ins | -0.00085 | -0.00042 | -0.00005 | 0.00356 | 0.00079 | 0.00368 | 0.00000 | 0.00150 | 0.00063 | -0.00158 | 0.00001 | 0.00220 | 0.00005 | -0.00290 |
| trg | -0.00152 | -0.00115 | -0.00158 | -0.00531 | -0.00263 | -0.00607 | 0.00150 | 0.00000 | -0.01497 | -0.01395 | 0.00008 | 0.00262 | 0.00011 | -0.00243 |
| ldl | -0.00242 | -0.00141 | -0.00390 | 0.00277 | -0.00049 | -0.00318 | 0.00063 | -0.01497 | 0.00000 | 0.00876 | 0.00043 | 0.02587 | 0.00011 | -0.01095 |
| hdl | 0.00041 | -0.00238 | 0.00029 | 0.00646 | 0.00749 | 0.02154 | -0.00158 | -0.01395 | 0.00876 | 0.00000 | 0.00042 | 0.00599 | -0.00010 | 0.01000 |
| age | -0.00042 | -0.00034 | -0.00035 | 0.00008 | 0.00004 | 0.00012 | 0.00001 | 0.00008 | 0.00043 | 0.00042 | 0.00000 | 0.00108 | 0.00102 | -0.00011 |
| fsh | -0.00335 | 0.00074 | -0.00258 | 0.00543 | -0.00003 | -0.00354 | 0.00220 | 0.00262 | 0.02587 | 0.00599 | 0.00108 | 0.00000 | 0.00061 | -0.03101 |
| mns | 0.00051 | 0.00064 | 0.00043 | 0.00017 | 0.00005 | 0.00014 | 0.00005 | 0.00011 | 0.00011 | -0.00010 | 0.00102 | 0.00061 | 0.00000 | -0.00001 |
| est | 0.00085 | 0.00021 | 0.00090 | 0.00212 | 0.00208 | 0.00275 | -0.00290 | -0.00243 | -0.01095 | 0.01000 | -0.00011 | -0.03101 | -0.00001 | 0.00000 |
|  |  |  |  |  |  |  |  |  |  |  |  |  |  |  |

*Note.* age = age at unique time-point; crp = C-reactive protein; dep = depressed mood; est = estradiol; fsh = follicle-stimulating hormones; fbr = fibrinogen; glc = fasting glucose; GVAR = graphical vector autoregressive models; hdl = high density lipoprotein; ins = insulin; int = interpersonal problems; lip = lipid marker composite; ldl = low density lipoprotein; mns = menopausal status; som = somatic symptoms; trg = triglycerides. Lower triangle indicates estimated partial contemporaneous associations, upper triangle indicates model-implied contemporaneous associations.

Table S2 (*continued*)

*Correlation Statistics of Panel GVAR Networks*

1. Between-Person Networks

|  | dep | som | int | fbr | crp | glc | ins | trg | ldl | hdl | age | fsh | mns | est |
| --- | --- | --- | --- | --- | --- | --- | --- | --- | --- | --- | --- | --- | --- | --- |
| dep | 0.00000 | 0.02111 | 0.02014 | 0.00064 | 0.00082 | 0.00157 | 0.00061 | 0.00059 | 0.00167 | -0.00270 | -0.00359 | -0.00065 | 0.00428 | -0.00004 |
| som | 0.02111 | 0.00000 | 0.01667 | 0.00143 | 0.00123 | 0.00179 | 0.00078 | 0.00073 | 0.00153 | -0.00341 | -0.00288 | -0.00066 | 0.00538 | 0.00001 |
| int | 0.02014 | 0.01667 | 0.00000 | 0.00079 | 0.00057 | 0.00103 | 0.00047 | 0.00041 | 0.00083 | -0.00213 | -0.00292 | -0.00047 | 0.00357 | 0.00011 |
| fbr | 0.00064 | 0.00143 | 0.00079 | 0.00000 | 0.00315 | 0.00137 | 0.00076 | 0.00041 | 0.00216 | -0.00344 | 0.00068 | -0.00030 | 0.00158 | -0.00022 |
| crp | 0.00082 | 0.00123 | 0.00057 | 0.00315 | 0.00000 | 0.00128 | 0.00059 | 0.00038 | 0.00026 | -0.00200 | 0.00029 | -0.00070 | 0.00046 | -0.00017 |
| glc | 0.00157 | 0.00179 | 0.00103 | 0.00137 | 0.00128 | 0.00000 | 0.00101 | 0.00100 | 0.00090 | -0.00257 | 0.00098 | -0.00038 | 0.00124 | -0.00022 |
| ins | 0.00061 | 0.00078 | 0.00047 | 0.00076 | 0.00059 | 0.00101 | 0.00000 | 0.00047 | 0.00017 | -0.00141 | 0.00018 | -0.00033 | 0.00050 | -0.00011 |
| trg | 0.00059 | 0.00073 | 0.00041 | 0.00041 | 0.00038 | 0.00100 | 0.00047 | 0.00000 | 0.00087 | -0.00312 | 0.00086 | -0.00015 | 0.00107 | -0.00024 |
| ldl | 0.00167 | 0.00153 | 0.00083 | 0.00216 | 0.00026 | 0.00090 | 0.00017 | 0.00087 | 0.00000 | -0.00446 | 0.00381 | 0.00068 | 0.00102 | -0.00030 |
| hdl | -0.00270 | -0.00341 | -0.00213 | -0.00344 | -0.00200 | -0.00257 | -0.00141 | -0.00312 | -0.00446 | 0.00000 | 0.00372 | 0.00310 | -0.00099 | 0.00055 |
| age | -0.00359 | -0.00288 | -0.00292 | 0.00068 | 0.00029 | 0.00098 | 0.00018 | 0.00086 | 0.00381 | 0.00372 | 0.00000 | 0.00994 | 0.00971 | -0.00124 |
| fsh | -0.00065 | -0.00066 | -0.00047 | -0.00030 | -0.00070 | -0.00038 | -0.00033 | -0.00015 | 0.00068 | 0.00310 | 0.00994 | 0.00000 | 0.00567 | -0.00059 |
| mns | 0.00428 | 0.00538 | 0.00357 | 0.00158 | 0.00046 | 0.00124 | 0.00050 | 0.00107 | 0.00102 | -0.00099 | 0.00971 | 0.00567 | 0.00000 | -0.00017 |
| est | -0.00004 | 0.00001 | 0.00011 | -0.00022 | -0.00017 | -0.00022 | -0.00011 | -0.00024 | -0.00030 | 0.00055 | -0.00124 | -0.00059 | -0.00017 | 0.00000 |
|  |  |  |  |  |  |  |  |  |  |  |  |  |  |  |

*Note.* age = age at unique time-point; crp = C-reactive protein; dep = depressed mood; est = estradiol; fsh = follicle-stimulating hormones; fbr = fibrinogen; glc = fasting glucose; GVAR = graphical vector autoregressive models; hdl = high density lipoprotein; ins = insulin; int = interpersonal problems; lip = lipid marker composite; ldl = low density lipoprotein; mns = menopausal status; som = somatic symptoms; trg = triglycerides. Lower triangle indicates estimated partial between-person associations, upper triangle indicates model-implied between-person associations.

Table S3

*Frequency of Each Network Edge Included in the 1000 Bootstrap Samples*

1. Within-Person Temporal Networks

|  | dep | som | int | fbr | crp | glc | ins | trg | ldl | hdl | age | fsh | mns | est |
| --- | --- | --- | --- | --- | --- | --- | --- | --- | --- | --- | --- | --- | --- | --- |
| dep | **1000** | 1 | 0 | **899** | **531** | **676** | **811** | 150 | **778** | 399 | **500** | 49 | **957** | **930** |
| som | 0 | **1000** | 3 | **995** | **908** | **644** | **816** | **801** | **699** | **876** | **500** | **746** | **939** | **703** |
| int | 0 | 231 | **1000** | **975** | 89 | 279 | 139 | 154 | **907** | **532** | 499 | **724** | **935** | **748** |
| fbr | 310 | **946** | 42 | **1000** | 0 | 0 | **998** | **1000** | **998** | 0 | **505** | **1000** | **936** | 0 |
| crp | **883** | **948** | **653** | 0 | **1000** | 49 | **998** | **1000** | **838** | 0 | 466 | **997** | **666** | 0 |
| glc | 64 | 462 | 3 | 0 | 0 | **1000** | **998** | **1000** | **1000** | 0 | **511** | **1000** | **869** | 0 |
| ins | 395 | 6 | 252 | 0 | **999** | 0 | **1000** | **999** | **999** | 1 | **503** | **1000** | **953** | 0 |
| trg | 488 | **592** | **537** | 256 | **883** | 0 | 2 | **1000** | **1000** | **1000** | 499 | **688** | **973** | **988** |
| ldl | **901** | 347 | **777** | 3 | **950** | 2 | **762** | **1000** | **1000** | 0 | 491 | 440 | **946** | 8 |
| hdl | **961** | **988** | **804** | **783** | 0 | **620** | **998** | **1000** | 0 | **1000** | 492 | **1000** | **528** | 0 |
| age | 380 | 466 | 295 | **851** | **934** | **844** | **803** | **906** | **1000** | **1000** | **1000** | **1000** | **998** | 5 |
| fsh | 219 | 9 | **605** | **976** | **1000** | 0 | **952** | **1000** | **998** | **600** | **535** | **1000** | **969** | 0 |
| mns | **904** | **879** | **864** | **909** | **625** | **961** | **933** | **766** | 155 | **498** | **1000** | **1000** | **1000** | 102 |
| est | **693** | **990** | **824** | **1000** | **991** | **1000** | **998** | 1 | 48 | **1000** | **534** | **949** | **972** | **1000** |
|  |  |  |  |  |  |  |  |  |  |  |  |  |  |  |

*Note.* age = age at unique time-point; crp = C-reactive protein; dep = depressed mood; est = estradiol; fsh = follicle-stimulating hormones; fbr = fibrinogen; glc = fasting glucose; hdl = high density lipoprotein; ins = insulin; int = interpersonal problems; lip = lipid marker composite; ldl = low density lipoprotein; mns = menopausal status; som = somatic symptoms; trg = triglycerides. This table shows frequency of estimates of partial directed associations included across all 1,000 bootstrap samples. Diagonal numbers indicate frequency of autocorrelations included across all 1,000 bootstrap samples. Lower triangle indicates frequency of estimates of variables in the leftmost column predicting future lag-1 variables in the topmost row included across all 1,000 bootstrap samples. Estimates in the upper triangle indicates the opposite temporal (lag-1) relations included across all 1,000 bootstrap samples. Numbers in grey shaded cells represent cross-lagged relations most important to our study aims.

Table S3 (*continued*)

*Frequency of Each Network Edge Included in the 1000 Bootstrap Samples*

1. Within-Person Contemporaneous Networks

|  | dep | som | int | fbr | crp | glc | ins | trg | ldl | hdl | age | fsh | mns | est |
| --- | --- | --- | --- | --- | --- | --- | --- | --- | --- | --- | --- | --- | --- | --- |
| dep | 0 | **1000** | **1000** | 127 | 68 | **718** | 71 | 36 | 73 | **627** | 0 | 13 | **1000** | **747** |
| som | **1000** | 0 | **1000** | 347 | 58 | **929** | 250 | 79 | 196 | 33 | 1 | **691** | **1000** | **562** |
| int | **1000** | **1000** | 0 | **721** | 162 | **983** | 459 | 29 | 8 | **589** | 0 | 44 | **1000** | **738** |
| fbr | 127 | 347 | **721** | 0 | **1000** | **1000** | **1000** | 0 | **985** | **1000** | **912** | **1000** | **998** | **988** |
| crp | 68 | 58 | 162 | **1000** | 0 | **1000** | **996** | 0 | 293 | **1000** | **867** | 488 | **926** | **998** |
| glc | **718** | **929** | **983** | **1000** | **1000** | 0 | **1000** | 0 | 3 | **1000** | **990** | 0 | **996** | **996** |
| ins | 71 | 250 | 459 | **1000** | **996** | **1000** | 0 | **998** | **912** | 0 | **789** | **1000** | **995** | 0 |
| trg | 36 | 79 | 29 | 0 | 0 | 0 | **998** | 0 | 0 | 0 | **996** | **1000** | **1000** | 0 |
| ldl | 73 | 196 | 8 | **985** | 293 | 3 | **912** | 0 | 0 | **1000** | **1000** | **1000** | **907** | 0 |
| hdl | **627** | 33 | **589** | **1000** | **1000** | **1000** | 0 | 0 | **1000** | 0 | **1000** | **1000** | 132 | **1000** |
| age | 0 | 1 | 0 | **912** | **867** | **990** | **789** | **996** | **1000** | **1000** | 0 | **1000** | **1000** | 1 |
| fsh | 13 | **691** | 44 | **1000** | 488 | 0 | **1000** | **1000** | **1000** | **1000** | **1000** | 0 | **1000** | 0 |
| mns | **1000** | **1000** | **1000** | **998** | **926** | **996** | **995** | **1000** | **907** | 132 | **1000** | **1000** | 0 | 410 |
| est | **747** | **562** | **738** | **988** | **998** | **996** | 0 | 0 | 0 | **1000** | 1 | 0 | 410 | 0 |
|  |  |  |  |  |  |  |  |  |  |  |  |  |  |  |

*Note.* age = age at unique time-point; crp = C-reactive protein; dep = depressed mood; est = estradiol; fsh = follicle-stimulating hormones; fbr = fibrinogen; glc = fasting glucose; hdl = high density lipoprotein; ins = insulin; int = interpersonal problems; lip = lipid marker composite; ldl = low density lipoprotein; mns = menopausal status; som = somatic symptoms; trg = triglycerides. Lower triangle indicates frequency of estimated partial contemporaneous associations included across all 1,000 bootstrap samples. Upper triangle indicates frequency of model-implied contemporaneous associations included across all 1,000 bootstrap samples. Estimates triangle indicates the opposite temporal (lag-1) relations included across all 1,000 bootstrap samples. Numbers in grey shaded cells represent cross-lagged relations most important to our study aims.

Table S3 (*continued*)

*Frequency of Each Network Edge Included in the 1000 Bootstrap Samples*

1. Between-Person Networks

|  | dep | som | int | fbr | crp | glc | ins | trg | ldl | hdl | age | fsh | mns | est |
| --- | --- | --- | --- | --- | --- | --- | --- | --- | --- | --- | --- | --- | --- | --- |
| dep | 0 | **1000** | **1000** | **972** | **1000** | **1000** | **1000** | **1000** | **1000** | 0 | 0 | 23 | **1000** | 387 |
| som | **1000** | 0 | **1000** | **1000** | **1000** | **1000** | **1000** | **1000** | **1000** | 0 | 1 | 25 | **1000** | **552** |
| int | **1000** | **1000** | 0 | **992** | **999** | **1000** | **1000** | **998** | **982** | 0 | 0 | 67 | **1000** | **801** |
| fbr | **972** | **1000** | **992** | 0 | **1000** | **1000** | **1000** | **1000** | **1000** | 0 | **890** | 38 | **998** | 4 |
| crp | **1000** | **1000** | **999** | **1000** | 0 | **1000** | **1000** | **1000** | **969** | 0 | **839** | 0 | **923** | 0 |
| glc | **1000** | **1000** | **1000** | **1000** | **1000** | 0 | **1000** | **1000** | **1000** | 0 | **990** | 8 | **995** | 1 |
| ins | **1000** | **1000** | **1000** | **1000** | **1000** | **1000** | 0 | **1000** | **954** | 0 | **860** | 0 | **998** | 0 |
| trg | **1000** | **1000** | **998** | **1000** | **1000** | **1000** | **1000** | 0 | **1000** | 0 | **999** | 72 | **1000** | 0 |
| ldl | **1000** | **1000** | **982** | **1000** | **969** | **1000** | **954** | **1000** | 0 | 0 | **1000** | **999** | **911** | 3 |
| hdl | 0 | 0 | 0 | 0 | 0 | 0 | 0 | 0 | 0 | 0 | **1000** | **1000** | 123 | **1000** |
| age | 0 | 1 | 0 | **890** | **839** | **990** | **860** | **999** | **1000** | **1000** | 0 | **1000** | **1000** | 0 |
| fsh | 23 | 25 | 67 | 38 | 0 | 8 | 0 | 72 | **999** | **1000** | **1000** | 0 | **1000** | 0 |
| mns | **1000** | **1000** | **1000** | **998** | **923** | **995** | **998** | **1000** | **911** | 123 | **1000** | **1000** | 0 | 236 |
| est | 387 | **552** | **801** | 4 | 0 | 1 | 0 | 0 | 3 | **1000** | 0 | 0 | 236 | 0 |
|  |  |  |  |  |  |  |  |  |  |  |  |  |  |  |

*Note.* age = age at unique time-point; crp = C-reactive protein; dep = depressed mood; est = estradiol; fsh = follicle-stimulating hormones; fbr = fibrinogen; glc = fasting glucose; hdl = high density lipoprotein; ins = insulin; int = interpersonal problems; lip = lipid marker composite; ldl = low density lipoprotein; mns = menopausal status; som = somatic symptoms; trg = triglycerides. Lower triangle indicates frequency of estimated partial between-person associations across all 1,000 bootstrap samples. Upper triangle indicates frequency of model-implied between-person associations across all 1,000 bootstrap samples. Numbers in grey shaded cells represent cross-lagged relations most important to our study aims.

Table S4

*Directed Edges of Within-Person Temporal (Lag-1) Network*

| Node-Out | Node-In | Edge | *p* | *d* |  | Node-Out | Node-In | Edge | *p* | *d* |
| --- | --- | --- | --- | --- | --- | --- | --- | --- | --- | --- |
| dep | dep | 0.08733 | 0.000 | 5.960 |  | int | dep | -0.01062 | 0.000 | -1.402 |
| dep | som | -0.00898 | 0.000 | -0.214 |  | int | som | -0.00161 | 0.000 | -0.039 |
| dep | int | -0.00957 | 0.000 | -3.214 |  | int | int | 0.07909 | 0.000 | 72.659 |
| dep | fbr | 0.00197 | 0.000 | 0.081 |  | **int** | **fbr** | **0.00284** | **0.000** | **0.129** |
| dep | crp | 0.00012 | 0.000 | 0.004 |  | int | crp | -0.00159 | 0.000 | -0.058 |
| dep | glc | 0.00075 | 0.000 | 0.029 |  | int | glc | -0.00086 | 0.000 | -0.036 |
| dep | ins | 0.00090 | 0.000 | 0.008 |  | int | ins | -0.00096 | 0.000 | -0.006 |
| dep | trg | -0.00091 | 0.000 | -0.009 |  | int | trg | -0.00105 | 0.000 | -0.011 |
| dep | ldl | 0.00131 | 0.000 | 0.094 |  | **int** | **ldl** | **0.00236** | **0.000** | **0.331** |
| dep | hdl | -0.00031 | 0.000 | -0.008 |  | int | hdl | 0.00009 | 0.000 | 0.003 |
| dep | age | 0.00000 | 0.000 | 0.000 |  | int | age | 0.00000 | 0.000 | 0.000 |
| dep | fsh | -0.00211 | 0.000 | -0.021 |  | int | fsh | 0.00090 | 0.000 | 0.024 |
| dep | mns | 0.00032 | 0.254 | 0.000 |  | int | mns | 0.00027 | 0.309 | 0.000 |
| dep | est | 0.00187 | 0.000 | 0.045 |  | int | est | 0.00089 | 0.000 | 0.023 |
| som | dep | -0.01056 | 0.000 | -0.288 |  | fbr | dep | -0.00054 | 0.000 | -0.076 |
| som | som | 0.08254 | 0.000 | 4.082 |  | **fbr** | **som** | **0.00166** | **0.000** | **0.156** |
| som | int | -0.00587 | 0.000 | -0.332 |  | fbr | int | -0.00172 | 0.000 | -0.365 |
| **som** | **fbr** | **0.00388** | **0.000** | **0.188** |  | fbr | fbr | 0.10238 | 0.000 | 75.100 |
| som | crp | 0.00164 | 0.000 | 0.056 |  | fbr | crp | -0.00839 | 0.000 | -1.197 |
| som | glc | 0.00061 | 0.000 | 0.025 |  | fbr | glc | -0.00640 | 0.000 | -1.010 |
| som | ins | 0.00074 | 0.000 | 0.009 |  | fbr | ins | 0.00605 | 0.000 | 0.522 |
| som | trg | 0.00064 | 0.000 | 0.006 |  | fbr | trg | 0.00437 | 0.000 | 0.442 |
| som | ldl | 0.00087 | 0.000 | 0.099 |  | fbr | ldl | 0.00365 | 0.000 | 0.393 |
| som | hdl | 0.00157 | 0.000 | 0.046 |  | fbr | hdl | -0.01005 | 0.000 | -1.085 |
| som | age | 0.00000 | 0.000 | 0.000 |  | fbr | age | 0.00000 | 0.000 | 0.000 |
| som | fsh | 0.00097 | 0.000 | 0.019 |  | fbr | fsh | 0.00860 | 0.000 | 0.910 |
| som | mns | 0.00032 | 0.245 | 0.000 |  | fbr | mns | 0.00024 | 0.634 | 0.000 |
| som | est | 0.00063 | 0.000 | 0.016 |  | fbr | est | -0.01544 | 0.000 | -1.437 |
|  |  |  |  |  |  |  |  |  |  |  |

Table S4 (*continued*)

*Directed Edges of Within-Person Temporal (Lag-1) Network*

| Node-Out | Node-In | Edge | *p* | *d* |  | Node-Out | Node-In | Edge | *p* | *d* |
| --- | --- | --- | --- | --- | --- | --- | --- | --- | --- | --- |
| **crp** | **dep** | **0.00081** | **0.000** | **1.072** |  | ins | dep | -0.00009 | 0.000 | -0.245 |
| **crp** | **som** | **0.00110** | **0.000** | **1.812** |  | ins | som | -0.00135 | 0.000 | -6.245 |
| **crp** | **int** | **0.00033** | **0.000** | **2.112** |  | ins | int | -0.00033 | 0.000 | -0.708 |
| crp | fbr | -0.00330 | 0.000 | -3.728 |  | ins | fbr | -0.00205 | 0.000 | -14.831 |
| crp | crp | 0.10073 | 0.000 | 34.744 |  | ins | crp | 0.00084 | 0.000 | 3.447 |
| crp | glc | -0.00128 | 0.000 | -1.381 |  | ins | glc | -0.00497 | 0.000 | -20.615 |
| crp | ins | 0.00465 | 0.000 | 6.532 |  | ins | ins | 0.11098 | 0.000 | 36.891 |
| crp | trg | 0.00166 | 0.000 | 4.031 |  | ins | trg | 0.00100 | 0.000 | 2.661 |
| crp | ldl | 0.00066 | 0.000 | 11.517 |  | ins | ldl | 0.00127 | 0.000 | 11.108 |
| crp | hdl | -0.00752 | 0.000 | -6.525 |  | ins | hdl | -0.00159 | 0.000 | -5.287 |
| crp | age | 0.00000 | 0.000 | 0.000 |  | ins | age | 0.00000 | 0.000 | 0.000 |
| crp | fsh | 0.00143 | 0.000 | 1.043 |  | ins | fsh | 0.00236 | 0.000 | 4.358 |
| crp | mns | 0.00004 | 0.104 | 0.000 |  | ins | mns | 0.00007 | 0.000 | 0.000 |
| crp | est | -0.00801 | 0.000 | -6.161 |  | ins | est | -0.00212 | 0.000 | -5.685 |
| glc | dep | -0.00201 | 0.000 | -0.289 |  | trg | dep | 0.00000 | 0.000 | -0.004 |
| glc | som | -0.00006 | 0.000 | -0.005 |  | **trg** | **som** | **0.00014** | **0.000** | **0.131** |
| glc | int | -0.00345 | 0.000 | -0.715 |  | trg | int | 0.00007 | 0.000 | 0.071 |
| glc | fbr | -0.00660 | 0.000 | -2.006 |  | trg | fbr | -0.00039 | 0.000 | -0.399 |
| glc | crp | -0.01402 | 0.000 | -2.745 |  | trg | crp | 0.00063 | 0.000 | 0.556 |
| glc | glc | 0.09628 | 0.000 | 11.065 |  | trg | glc | -0.00264 | 0.000 | -2.434 |
| glc | ins | 0.01050 | 0.000 | 2.129 |  | trg | ins | -0.00220 | 0.000 | -0.830 |
| glc | trg | 0.00557 | 0.000 | 0.688 |  | trg | trg | 0.10500 | 0.000 | 1.764 |
| glc | ldl | 0.00702 | 0.000 | 0.775 |  | trg | ldl | 0.00587 | 0.000 | 10.165 |
| glc | hdl | -0.01811 | 0.000 | -2.650 |  | trg | hdl | 0.00222 | 0.000 | 1.431 |
| glc | age | 0.00000 | 0.000 | 0.000 |  | trg | age | 0.00000 | 0.000 | 0.000 |
| glc | fsh | 0.01294 | 0.000 | 1.254 |  | trg | fsh | 0.00024 | 0.000 | 0.083 |
| glc | mns | 0.00012 | 0.802 | 0.000 |  | trg | mns | 0.00030 | 0.000 | 0.000 |
| glc | est | -0.02157 | 0.000 | -2.355 |  | trg | est | 0.00089 | 0.000 | 0.527 |
|  |  |  |  |  |  |  |  |  |  |  |

Table S4 (*continued*)

*Directed Edges of Within-Person Temporal (Lag-1) Network*

| Node-Out | Node-In | Edge | *p* | *d* |  | Node-Out | Node-In | Edge | *p* | *d* |
| --- | --- | --- | --- | --- | --- | --- | --- | --- | --- | --- |
| **ldl** | **dep** | **0.00155** | **0.000** | **0.251** |  | **hdl** | **dep** | **0.00172** | **0.000** | **0.196** |
| ldl | som | -0.00043 | 0.000 | -0.074 |  | **hdl** | **som** | **0.00214** | **0.000** | **0.162** |
| **ldl** | **int** | **0.00101** | **0.000** | **0.327** |  | **hdl** | **int** | **0.00082** | **0.000** | **0.134** |
| ldl | fbr | -0.00270 | 0.000 | -0.229 |  | hdl | fbr | 0.00054 | 0.000 | 0.167 |
| ldl | crp | 0.00141 | 0.000 | 0.101 |  | hdl | crp | -0.00629 | 0.000 | -2.187 |
| ldl | glc | -0.00291 | 0.000 | -0.224 |  | hdl | glc | 0.00027 | 0.000 | 0.060 |
| ldl | ins | 0.00050 | 0.000 | 0.015 |  | hdl | ins | 0.00434 | 0.000 | 0.511 |
| ldl | trg | 0.01114 | 0.000 | 0.625 |  | hdl | trg | 0.00235 | 0.000 | 0.225 |
| ldl | ldl | 0.09496 | 0.000 | 10.134 |  | hdl | ldl | -0.00440 | 0.000 | -0.459 |
| ldl | hdl | -0.00666 | 0.000 | -0.375 |  | hdl | hdl | 0.10059 | 0.000 | 8.627 |
| ldl | age | 0.00000 | 0.000 | 0.000 |  | hdl | age | 0.00000 | 0.000 | 0.000 |
| ldl | fsh | -0.00014 | 0.000 | -0.006 |  | hdl | fsh | 0.00336 | 0.000 | 0.348 |
| ldl | mns | 0.00036 | 0.356 | 0.000 |  | hdl | mns | 0.00000 | 0.996 | 0.000 |
| ldl | est | -0.00216 | 0.000 | -0.102 |  | hdl | est | -0.00857 | 0.000 | -0.658 |
|  |  |  |  |  |  |  |  |  |  |  |

*Note.* age = age at unique time-point; crp = C-reactive protein; dep = depressed mood; est = estradiol; fsh = follicle-stimulating hormones; fbr = fibrinogen; glc = fasting glucose; hdl = high density lipoprotein; ins = insulin; int = interpersonal problems; lip = lipid marker composite; ldl = low density lipoprotein; mns = menopausal status; som = somatic symptoms; trg = triglycerides. Bold values reflect statistically significant edges. Parameter estimates in grey shaded cells represent cross-lagged relations most important to our study aims.

Table S5

*Undirected Edges of Within-Person Contemporaneous Network*

| Node-Out | Node-In | Edge | *p* | *d* |  | Node-Out | Node-In | Edge | *p* | *d* |
| --- | --- | --- | --- | --- | --- | --- | --- | --- | --- | --- |
| dep | som | 0.11766 | 0.000 | 4.212 |  | int | ins | -0.00002 | 0.000 | -0.003 |
| dep | int | 0.11748 | 0.000 | 1.984 |  | int | trg | -0.00153 | 0.000 | -0.302 |
| dep | fbr | -0.00152 | 0.000 | -0.158 |  | int | ldl | -0.00399 | 0.000 | -0.459 |
| dep | crp | -0.00156 | 0.000 | -0.381 |  | **int** | **hdl** | **0.00033** | **0.000** | **0.181** |
| **dep** | **glc** | **0.00084** | **0.000** | **0.298** |  | int | age | -0.00034 | 0.000 | -0.063 |
| dep | ins | -0.00083 | 0.000 | -0.091 |  | int | fsh | -0.00258 | 0.000 | -0.129 |
| dep | trg | -0.00144 | 0.000 | -0.909 |  | int | mns | 0.00044 | 0.000 | 0.000 |
| dep | ldl | -0.00246 | 0.000 | -0.526 |  | int | est | 0.00101 | 0.000 | 0.442 |
| dep | hdl | 0.00042 | 0.000 | 0.096 |  | fbr | crp | 0.03411 | 0.000 | 76.546 |
| dep | age | -0.00043 | 0.000 | -0.071 |  | fbr | glc | 0.03807 | 0.000 | 32.069 |
| dep | fsh | -0.00334 | 0.000 | -0.234 |  | fbr | ins | 0.00352 | 0.000 | 17.500 |
| dep | mns | 0.00052 | 0.000 | 0.000 |  | fbr | trg | -0.00527 | 0.000 | -7.494 |
| dep | est | 0.00096 | 0.000 | 0.129 |  | fbr | ldl | 0.00273 | 0.000 | 0.575 |
| som | int | 0.07645 | 0.000 | 1.988 |  | fbr | hdl | 0.00645 | 0.000 | 7.853 |
| som | fbr | -0.00052 | 0.000 | -0.526 |  | fbr | age | 0.00009 | 0.000 | 0.004 |
| som | crp | -0.00147 | 0.000 | -1.288 |  | fbr | fsh | 0.00539 | 0.000 | 0.484 |
| **som** | **glc** | **0.00200** | **0.000** | **3.586** |  | fbr | mns | 0.00017 | 0.000 | 0.000 |
| som | ins | -0.00041 | 0.000 | -0.107 |  | fbr | est | 0.00212 | 0.000 | 2.065 |
| som | trg | -0.00107 | 0.000 | -0.224 |  | crp | glc | 0.02526 | 0.000 | 31.406 |
| som | ldl | -0.00144 | 0.000 | -0.175 |  | crp | ins | 0.00075 | 0.000 | 3.676 |
| som | hdl | -0.00242 | 0.000 | -0.521 |  | crp | trg | -0.00261 | 0.000 | -5.475 |
| som | age | -0.00034 | 0.000 | -0.059 |  | crp | ldl | -0.00050 | 0.000 | -2.263 |
| som | fsh | 0.00080 | 0.000 | 0.058 |  | crp | hdl | 0.00746 | 0.000 | 43.487 |
| som | mns | 0.00064 | 0.000 | 0.000 |  | crp | age | 0.00004 | 0.000 | 0.020 |
| som | est | 0.00031 | 0.000 | 0.094 |  | crp | fsh | -0.00005 | 0.000 | -0.132 |
| **int** | **fbr** | **0.00095** | **0.000** | **1.029** |  | crp | mns | 0.00005 | 0.000 | 0.000 |
| int | crp | -0.00097 | 0.000 | -1.011 |  | crp | est | 0.00208 | 0.000 | 7.762 |
| **int** | **glc** | **0.00314** | **0.000** | **1.055** |  | glc | ins | 0.00365 | 0.000 | 16.738 |
|  |  |  |  |  |  |  |  |  |  |  |

Table S5 (*continued*)

*Undirected Edges of Within-Person Contemporaneous Network*

| Node-Out | Node-In | Edge | *p* | *d* |  | Node-Out | Node-In | Edge | *p* | *d* |
| --- | --- | --- | --- | --- | --- | --- | --- | --- | --- | --- |
| glc | trg | -0.00602 | 0.000 | -7.767 |  | ldl | hdl | 0.00875 | 0.000 | 5.772 |
| glc | ldl | -0.00317 | 0.000 | -0.839 |  | ldl | age | 0.00043 | 0.000 | 0.036 |
| glc | hdl | 0.02147 | 0.000 | 29.258 |  | ldl | fsh | 0.02590 | 0.000 | 2.406 |
| glc | age | 0.00012 | 0.000 | 0.007 |  | ldl | mns | 0.00011 | 0.000 | 0.000 |
| glc | fsh | -0.00360 | 0.000 | -0.554 |  | ldl | est | -0.01098 | 0.000 | -7.080 |
| glc | mns | 0.00014 | 0.000 | 0.000 |  | hdl | age | 0.00041 | 0.000 | 0.016 |
| glc | est | 0.00277 | 0.000 | 2.135 |  | hdl | fsh | 0.00598 | 0.000 | 2.357 |
| ins | trg | 0.00149 | 0.000 | 2.335 |  | hdl | mns | -0.00011 | 0.000 | 0.000 |
| ins | ldl | 0.00062 | 0.000 | 1.630 |  | hdl | est | 0.00999 | 0.000 | 4.873 |
| ins | hdl | -0.00159 | 0.000 | -4.979 |  | age | fsh | 0.00108 | 0.000 | 0.065 |
| ins | age | 0.00001 | 0.000 | 0.016 |  | age | mns | 0.00102 | 0.000 | 0.019 |
| ins | fsh | 0.00219 | 0.000 | 3.424 |  | age | est | -0.00011 | 0.000 | -0.004 |
| ins | mns | 0.00005 | 0.000 | 0.000 |  | fsh | mns | 0.00061 | 0.000 | 0.000 |
| ins | est | -0.00288 | 0.000 | -4.887 |  | fsh | est | -0.03098 | 0.000 | -33.253 |
| trg | ldl | -0.01499 | 0.000 | -15.637 |  | mns | est | -0.00001 | 0.318 | 0.000 |
| trg | hdl | -0.01389 | 0.000 | -6.303 |  |  |  |  |  |  |
| trg | age | 0.00008 | 0.000 | 0.074 |  |  |  |  |  |  |
| trg | fsh | 0.00261 | 0.000 | 0.983 |  |  |  |  |  |  |
| trg | mns | 0.00011 | 0.000 | 0.000 |  |  |  |  |  |  |
| trg | est | -0.00241 | 0.000 | -78.559 |  |  |  |  |  |  |
|  |  |  |  |  |  |  |  |  |  |  |

*Note.* age = age at unique time-point; crp = C-reactive protein; dep = depressed mood; est = estradiol; fsh = follicle-stimulating hormones; fbr = fibrinogen; glc = fasting glucose; hdl = high density lipoprotein; ins = insulin; int = interpersonal problems; lip = lipid marker composite; ldl = low density lipoprotein; mns = menopausal status; som = somatic symptoms; trg = triglycerides. Bold values reflect statistically significant edges. Parameter estimates in grey shaded cells represent cross-lagged relations most important to our study aims.

Table S6

*Undirected Edges of Between-Person Network*

| Node-Out | Node-In | Edge | *p* | *d* |  | Node-Out | Node-In | Edge | *p* | *d* |
| --- | --- | --- | --- | --- | --- | --- | --- | --- | --- | --- |
| dep | som | 0.0212 | 0.000 | 0.769 |  | int | ins | 0.0005 | 0.000 | 0.095 |
| dep | int | 0.0202 | 0.000 | 0.127 |  | int | trg | 0.0004 | 0.000 | 0.031 |
| dep | fbr | 0.0006 | 0.000 | 0.019 |  | int | ldl | 0.0008 | 0.000 | 0.017 |
| **dep** | **crp** | **0.0008** | **0.000** | **0.205** |  | int | hdl | -0.0021 | 0.000 | -0.018 |
| **dep** | **glc** | **0.0016** | **0.000** | **0.138** |  | int | age | -0.0029 | 0.000 | -0.069 |
| dep | ins | 0.0006 | 0.000 | 0.079 |  | int | fsh | -0.0005 | 0.000 | -0.003 |
| dep | trg | 0.0006 | 0.000 | 0.033 |  | int | mns | 0.0036 | 0.000 | 0.009 |
| dep | ldl | 0.0017 | 0.000 | 0.038 |  | int | est | 0.0001 | 0.000 | 0.013 |
| dep | hdl | -0.0027 | 0.000 | -0.018 |  | fbr | crp | 0.0032 | 0.000 | 0.600 |
| dep | age | -0.0036 | 0.000 | -0.064 |  | fbr | glc | 0.0014 | 0.000 | 0.104 |
| dep | fsh | -0.0006 | 0.129 | -0.001 |  | fbr | ins | 0.0008 | 0.000 | 0.929 |
| dep | mns | 0.0043 | 0.000 | 0.012 |  | fbr | trg | 0.0004 | 0.000 | 0.033 |
| dep | est | 0.0000 | 0.056 | -0.001 |  | fbr | ldl | 0.0022 | 0.000 | 0.035 |
| som | int | 0.0167 | 0.000 | 0.225 |  | fbr | hdl | -0.0034 | 0.000 | -0.299 |
| som | fbr | 0.0014 | 0.000 | 0.052 |  | fbr | age | 0.0007 | 0.000 | 0.009 |
| **som** | **crp** | **0.0012** | **0.000** | **0.240** |  | fbr | fsh | -0.0003 | 0.000 | -0.002 |
| **som** | **glc** | **0.0018** | **0.000** | **0.447** |  | fbr | mns | 0.0016 | 0.000 | 0.008 |
| **som** | **ins** | **0.0008** | **0.000** | **0.231** |  | fbr | est | -0.0002 | 0.000 | -0.042 |
| som | trg | 0.0007 | 0.000 | 0.021 |  | crp | glc | 0.0013 | 0.000 | 1.647 |
| som | ldl | 0.0015 | 0.000 | 0.085 |  | crp | ins | 0.0006 | 0.000 | 0.983 |
| som | hdl | -0.0034 | 0.000 | -0.027 |  | crp | trg | 0.0004 | 0.000 | 0.177 |
| som | age | -0.0029 | 0.000 | -0.115 |  | crp | ldl | 0.0003 | 0.000 | 0.030 |
| som | fsh | -0.0007 | 0.000 | -0.003 |  | crp | hdl | -0.0020 | 0.000 | -0.216 |
| som | mns | 0.0054 | 0.000 | 0.054 |  | crp | age | 0.0003 | 0.000 | 0.012 |
| som | est | 0.0000 | 0.050 | 0.001 |  | crp | fsh | -0.0007 | 0.000 | -0.055 |
| int | fbr | 0.0008 | 0.000 | 0.066 |  | crp | mns | 0.0005 | 0.000 | 0.119 |
| int | crp | 0.0006 | 0.000 | 0.066 |  | crp | est | -0.0002 | 0.000 | -0.033 |
| int | glc | 0.0010 | 0.000 | 0.044 |  | glc | ins | 0.0010 | 0.000 | 0.583 |
|  |  |  |  |  |  |  |  |  |  |  |

Table S6 (*continued*)

*Undirected Edges of Between-Person Network*

| Node-Out | Node-In | Edge | *p* | *d* |  | Node-Out | Node-In | Edge | *p* | *d* |
| --- | --- | --- | --- | --- | --- | --- | --- | --- | --- | --- |
| glc | trg | 0.0010 | 0.000 | 0.135 |  | ldl | hdl | -0.0045 | 0.000 | -0.032 |
| glc | ldl | 0.0009 | 0.000 | 0.010 |  | ldl | age | 0.0038 | 0.000 | 0.135 |
| glc | hdl | -0.0026 | 0.000 | -0.174 |  | ldl | fsh | 0.0007 | 0.000 | 0.003 |
| glc | age | 0.0010 | 0.000 | 0.007 |  | ldl | mns | 0.0010 | 0.000 | 0.031 |
| glc | fsh | -0.0004 | 0.000 | -0.002 |  | ldl | est | -0.0003 | 0.000 | -0.012 |
| glc | mns | 0.0012 | 0.000 | 0.010 |  | hdl | age | 0.0037 | 0.000 | 0.170 |
| glc | est | -0.0002 | 0.000 | -0.012 |  | hdl | fsh | 0.0031 | 0.000 | 0.029 |
| ins | trg | 0.0005 | 0.000 | 0.467 |  | hdl | mns | -0.0010 | 0.000 | -0.006 |
| ins | ldl | 0.0002 | 0.000 | 0.027 |  | hdl | est | 0.0006 | 0.000 | 0.007 |
| ins | hdl | -0.0014 | 0.000 | -0.132 |  | age | fsh | 0.0099 | 0.000 | 0.958 |
| ins | age | 0.0002 | 0.000 | 0.002 |  | age | mns | 0.0097 | 0.000 | 1.669 |
| ins | fsh | -0.0003 | 0.000 | -0.013 |  | age | est | -0.0012 | 0.000 | -0.021 |
| ins | mns | 0.0005 | 0.000 | 0.033 |  | fsh | mns | 0.0057 | 0.000 | 0.019 |
| ins | est | -0.0001 | 0.000 | -0.039 |  | fsh | est | -0.0006 | 0.000 | -0.008 |
| trg | ldl | 0.0009 | 0.000 | 0.022 |  | mns | est | -0.0002 | 0.008 | -0.001 |
| trg | hdl | -0.0031 | 0.000 | -0.164 |  |  |  |  |  |  |
| trg | age | 0.0009 | 0.000 | 0.038 |  |  |  |  |  |  |
| trg | fsh | -0.0001 | 0.019 | -0.001 |  |  |  |  |  |  |
| trg | mns | 0.0011 | 0.000 | 0.016 |  |  |  |  |  |  |
| trg | est | -0.0002 | 0.000 | -0.031 |  |  |  |  |  |  |
|  |  |  |  |  |  |  |  |  |  |  |

*Note.* age = age at unique time-point; crp = C-reactive protein; dep = depressed mood; est = estradiol; fsh = follicle-stimulating hormones; fbr = fibrinogen; glc = fasting glucose; hdl = high density lipoprotein; ins = insulin; int = interpersonal problems; lip = lipid marker composite; ldl = low density lipoprotein; mns = menopausal status; som = somatic symptoms; trg = triglycerides. Bold values reflect statistically significant edges. Parameter estimates in grey shaded cells represent cross-lagged relations most important to our study aims.

Figure S1

*Community Structure Detection with Walktrap Algorithm*

*Note.* Bio = surrogate immunometabolism markers; Cov = covariates; crp = C-reactive protein; dep = depressed mood; est = estradiol; fbr = fibrinogen; fsh = follicle-stimulating hormone; glc = glucose; hdl = high density lipoprotein; ins = insulin; int = interpersonal problems; lip = lipid marker composite; ldl = low density lipoprotein; mns = menopausal status; som = somatic symptoms; trg = triglycerides. All scores have been rescaled to range from 1 to 4 prior to network estimation.

Figure S2

1. *Confidence Intervals of Edges from Within-Person Temporal (Lag-1) Network*

*Note.* Bio = biomarkers; Cov = covariates; crp = C-reactive protein; dep = depressed mood; est = estradiol; fbr = fibrinogen; fsh = follicle-stimulating hormone; glc = glucose; hdl = high density lipoprotein; ins = insulin; int = interpersonal problems; lip = lipid marker composite; ldl = low density lipoprotein; mns = menopausal status; som = somatic symptoms; trg = triglycerides.

Figure S2

1. *Confidence Intervals of Edges from Within-Person Contemporaneous Network*

*Note.* Bio = biomarkers; Cov = covariates; crp = C-reactive protein; dep = depressed mood; est = estradiol; fbr = fibrinogen; fsh = follicle-stimulating hormone; glc = glucose; hdl = high density lipoprotein; ins = insulin; int = interpersonal problems; lip = lipid marker composite; ldl = low density lipoprotein; mns = menopausal status; som = somatic symptoms; trg = triglycerides.

Figure S2

1. *Confidence Intervals of Edges from Between-Person Network*

*Note.* Bio = biomarkers; Cov = covariates; crp = C-reactive protein; dep = depressed mood; est = estradiol; fbr = fibrinogen; fsh = follicle-stimulating hormone; glc = glucose; hdl = high density lipoprotein; ins = insulin; int = interpersonal problems; lip = lipid marker composite; ldl = low density lipoprotein; mns = menopausal status; som = somatic symptoms; trg = triglycerides.

References

Hamaker, E. L., Kuiper, R. M., & Grasman, R. P. P. P. (2015). A critique of the cross-lagged panel model. *Psychological Methods, 20*, 102-116. doi:10.1037/a0038889

Lee, T., & Shi, D. (2021). A comparison of full information maximum likelihood and multiple imputation in structural equation modeling with missing data. *Psychological Methods, 26*, 466-485. doi:10.1037/met0000381

van Buuren, S., & Groothuis-Oudshoorn, K. (2011). mice: Multivariate imputation by chained equations in R. *Journal of Statistical Software, 45*, 1548-7660.

Wild, B., Eichler, M., Friederich, H.-C., Hartmann, M., Zipfel, S., & Herzog, W. (2010). A graphical vector autoregressive modelling approach to the analysis of electronic diary data. *BMC Medical Research Methodology, 10*, 28. doi:10.1186/1471-2288-10-28
