# Supplementary material for: Prospective network analysis of proinflammatory proteins, lipid markers, and depression components in midlife community women
Source: Psychol Med. 2022 Aug 4;53(11):5267–78. doi: 10.1017/S003329172200232X (PMC9898473; doi:10.1017/S003329172200232X)
Supplement: Supplementary file 1 [file S003329172200232Xsup.zip › S003329172200232Xsup001.docx]

| Word count (Abstract): | 249 |
| --- | --- |
| Word count (Manuscript): | 4,500 |
| Tables: | 2 |
| Figures: | 3 |
| Supplementary Tables: | 6 |
| Supplementary Figures: | 2 |

Prospective Network Analysis of Proinflammatory Proteins, Lipid Markers, and Depression Components in Midlife Community Women

Nur Hani Zainal, M.S.

Harvard Medical School

Michelle G. Newman, Ph.D.

The Pennsylvania State University

**Author Note**

Please direct all correspondence to Dr. Michelle G. Newman ([mgn1@psu.edu](mailto:mgn1@psu.edu)). This article has not been peer-reviewed. Please do not cite without the authors’ permission.

Abstract

**Background:** Vulnerability theories propose that suboptimal levels of lipid markers and proinflammatory proteins predict future heightened depression. Scar models posit the reverse association. However, most studies that tested relationships between non-specific immune/endocrine markers and depression did not separate temporal inferences between people and within-person and how different immunometabolism markers related to unique depression symptoms. We thus used cross-lagged prospective network analyses (CLPN) to investigate this topic. **Methods:** Community midlife women (*n* = 2,224) completed the Center for Epidemiologic Studies-Depression scale and provided biomarker samples across five time-points spanning nine years. CLPN identified significant relations (*edges*) among components (*nodes*) of depression (depressed mood, somatic symptoms, interpersonal issues), lipid markers (insulin, fasting glucose, triglycerides, low-density lipoprotein-cholesterol (LDL), high-density lipoprotein-cholesterol (HDL)), and proinflammatory proteins (C-reactive protein (CRP), fibrinogen), within and across time-points. All models adjusted for age, estradiol, follicle-stimulating hormone, and menopausal status. **Results:** In within-person temporal networks, higher CRP and HDL predicted all three depression components (*d* = 0.131 to 2.112). Increased LDL preceded higher depressed mood and interpersonal issues (vs. somatic symptoms) (*d* = 0.251 to 0.327). Elevated triglycerides predicted more somatic symptoms (vs. depressed mood and interpersonal problems) (*d* = 0.131). More interpersonal problems forecasted elevated fibrinogen and LDL levels (*d* = 0.129 to 0.331), and stronger somatic symptoms preceded higher fibrinogen levels (*d* = 0.188). **Conclusions:** Results supported both vulnerability and scar models. Long-term dysregulated immunometabolism systems, social disengagement, and related patterns are possible mechanistic accounts. Cognitive-behavioral therapies that optimize nutrition and physical activity may effectively target depression.

*Keywords:* cross-lagged; network analysis; inflammation; immune; endocrine; depression; interpersonal; scar theory; vulnerability theory

Word count: 249

Heightened depression symptoms are commonly observed in the general population annually and across the lifetime (Jeuring et al., 2018). Reliable evidence has linked subthreshold depression to many physical ailments involving the cardiometabolic, gastrointestinal, and autoimmune systems (Simpson et al., 2021). Elevated depression also adversely affects romantic and professional relationships, career development, and other life satisfaction domains (Sivertsen, Bjorklof, Engedal, Selbaek, & Helvik, 2015). Economically, heightened depression consumes significant annual government expenditure (Revicki et al., 2012). Thus, a better understanding of the risk factors and consequences of elevated depression components is essential.

Our immune and endocrine systems dynamically interact with depressed mood and related symptoms by regulating the sympathetic nervous system, vagus nerve, hypothalamic-pituitary axis (HPA), and associated systems (Peirce & Alvina, 2019; Thayer & Fischer, 2009). These regulatory systems optimize inflammation levels to fend off infections, injuries, and toxins (Ellins, Rees, Deanfield, Steptoe, & Halcox, 2017). Two types of inflammation exist. Short-term (acute) inflammation is triggered by sugary and fatty substances, viruses, and bacteria that may result from sickness recovery, wound reparation, and brief stress episodes and is, on balance, adaptive (Cecconello, Clària Ribas, & Norling, 2022). Conversely, long-term (chronic) inflammation can build up plaques, clot the bloodstream and impair the brain, heart, and other organs (Michels, van Aart, Morisse, Mullee, & Huybrechts, 2021). Likewise, our endocrine system, which comprises glands that secrete and absorb hormones, lipids, and related markers, needs optimal balance to modulate mood states effectively (Chen et al., 2016). Prolonged inflammation and suboptimal lipid marker levels can thus contribute to autoimmune disorders, depressed mood, and associated symptoms by lowering resilience to stress and corresponding processes (Dedoncker, Vanderhasselt, Ottaviani, & Slavich, 2021; Suvarna et al., 2020).

Potential risk factors or consequences of elevated depression components have been theorized to include suboptimal levels of chronic peripheral proinflammatory proteins and lipid markers (e.g., Penninx, 2017). Proxy lipid markers might comprise unique hormones (e.g., insulin, fasting glucose), fats (e.g., triglycerides), and a combination of proteins and fats (e.g., low-density lipoprotein cholesterol (LDL), high-density lipoprotein cholesterol (HDL)) (Marz et al., 2017). C-reactive protein (CRP) and fibrinogen are acute-phase pro-inflammatory proteins the liver secretes in response to increased interleukin-6 (IL-6) and tends to be embedded in plasma and other bodily fluids (Johansson-Persson et al., 2014). CRP, in conjunction with damaged cells or sets of disease-producing microorganisms, primarily serves to activate adjunct systems (Macleod & Avery, 1941). Fibrinogen is a clotting agent precursor of the enzyme fibrin and is instrumental in platelet aggregation when fixing tissue and vascular injuries but contributes to heart problems in excessive amounts (Duivis et al., 2011). Depression components include somatic symptoms (e.g., appetite changes, sleep disturbances), depressed mood, and interpersonal problems (e.g., perceived unfriendliness) (Cosco, Prina, Stubbs, & Wu, 2017). These variables have been incorporated into vulnerability and scar theories of depression.

In particular, *vulnerability models* propose that suboptimal levels of insulin, fasting glucose, triglycerides, LDL, and HDL might influence future somatic (vs. mood and interpersonal) aspects of depression (Lamers et al., 2020; Penninx, 2017). Surrogate lipid markers have been theorized to predict future depressed mood and somatic symptoms through reduced neurogenesis, suboptimal cell metabolism function, and heightened inflammation (Dantzer, O'Connor, Freund, Johnson, & Kelley, 2008). These processes could decrease motivation to engage regularly in healthy behaviors and physical activity (Ignacio, da Silva, Plissari, Quevedo, & Reus, 2019), leading to subsequent depressed mood. Proinflammatory proteins that likely predict multiple aspects of depression include CRP and fibrinogen (Konsman, 2019; Lafitte et al., 2015). As they deplete dopaminergic neurons and disrupt mitochondrial function (e.g., glucose production) (Dantzer, Casaril, & Vichaya, 2021), increased proinflammatory proteins (vs. proxy lipid markers) would likely more strongly impact somatic (vs. mood and interpersonal) depression components (Majd, Saunders, & Engeland, 2020). Proinflammatory proteins (vs. proxy lipid markers) could, to a larger degree, perpetuate ‘sickness behaviors’ (i.e., fatigue, reduced activities) and negatively impact emotion regulation-related brain areas (e.g., dorsal anterior cingulate cortex, ventromedial prefrontal cortex) (Felger et al., 2016; Torres-Platas, Cruceanu, Chen, Turecki, & Mechawar, 2014), leading to future elevated somatic symptoms.

*Scar theories* posit that somatic symptoms, compared to depressed mood and interpersonal issues, are depression components with the most extensive relations to future increased proinflammatory proteins (Felger et al., 2020; Lamers et al., 2013) vs. surrogate lipid markers (Rotella & Mannucci, 2013). These processes could occur via the buildup of stress hormones and chronic dysregulation of the HPA over long periods (Dias et al., 2020; Vingeliene, Hiyoshi, Lentjes, Fall, & Montgomery, 2019). Suboptimal habits (e.g., decreased exercise, excessive caloric intake, or carbohydrate-dense foods) and social withdrawal patterns (Feng & Astell-Burt, 2017) could mediate elevated depression components predicting worse immunometabolism. Also, depression components might adversely affect immunometabolism via decreased attempts to tap into social support resources during stress (Gouin, Wrosch, McGrath, & Booij, 2020). Increased social isolation could negatively alter the body's reactivity toward biological or interpersonal stressors (e.g., worsening social cohesion) (Smith, Gavey, NE, Kontari, & Victor, 2020). These challenges could prompt more robust long-term increased proinflammatory (vs. proxy lipid markers) responses, resulting in more somatic symptoms relative to depressed mood and interpersonal issues (Smith et al., 2020).

Prospective data to date reliably support the theories above. Consistent with vulnerability and scar models, data across 15 studies showed that excessive surrogate lipid markers (e.g., insulin, fasting glucose) bidirectionally predicted future major depression severity and diagnosis in clinical and community samples (cf. meta-analysis and empirical study by Hiles, Revesz, Lamers, Giltay, & Penninx, 2016; Pan et al., 2012). Likewise, concordant with vulnerability models and scar theories, more than 85 studies that recruited diverse youth and adult populations showed that depressive symptoms bidirectionally predicted heightened surrogate lipid markers and non-specific proinflammatory proteins (e.g., IL-6, CRP, fibrinogen) across two months to 18 years (cf. reviews and empirical studies by Colasanto, Madigan, & Korczak, 2020; Lamers et al., 2019; Mac Giollabhui, Ng, Ellman, & Alloy, 2021; Valkanova, Ebmeier, & Allan, 2013; Zainal & Newman, 2021c, 2022). Collectively, suboptimal proinflammatory proteins and surrogate lipid marker levels could be bidirectionally related to somatic symptoms, depressed mood, and interpersonal problems. Moreover, the literature offers more evidence for vulnerability models than scar theories (e.g., Mac Giollabhui et al., 2021).

However, most prior longitudinal studies thus far have not tested how *components* of proinflammatory proteins, surrogate lipid markers, and depression related to one another. Examining these relationships is essential because depression may arise from the interactions among these mutually-influencing components, and unique depression components could relate differently in magnitude and direction to distinct surrogate immunometabolism markers (Zhang et al., 2022). In addition, the literature is replete with studies on this topic using ordinary least squares (OLS) regression and structural equation modeling (SEM) approaches. Although informative, OLS, SEM, and other traditional statistical approaches tend to yield parameters that enable understanding of the relations among the *mean-overall score or latent constructs* rather than relations among the *components* of these constructs. The latent variable modeling approach precludes determining unique immunometabolism trajectories for persons with the same mean-overall score but elevated on different components (e.g., high somatic symptoms and low depressed mood versus low somatic symptoms and high depressed mood). Cross-lagged prospective network analysis (CLPN) (Epskamp, 2020) is thus a means to understand how components (or *nodes*) rather than latent constructs relate to one another in a network of mutually-influencing nodes across multiple time-points, within and between persons. Relations between nodes are called *edges*, typically expressed as partial correlations that have adjusted for the effects of all other nodes. Moreover, CLPN permits identifying nodes with the biggest impact and the highest number of associations with all future nodes (Borsboom et al., 2021). These most impactful nodes in temporal networks are key therapy targets, as altering those influential nodes might change future depression nodes (Roefs et al., 2022).

To date, only six studies have used network analyses with cross-sectional data to investigate this topic. Recently, Jia et al. (2020) observed that although higher HDL levels coincided with stronger concurrent depressive symptoms, other lipid markers (e.g., triglycerides, LDL) had null relations. Further, depressive symptoms were nodes with the most robust connections with other nodes in the network (Jia et al., 2020). A separate network analysis showed that higher IL-6 and CRP more strongly coincided with increased somatic symptoms (i.e., aches, pains, sleep issues) versus other depression nodes in Dutch adults with and without elevated depression (Fried et al., 2020). Another network analysis found that persons with (vs. without) heightened CRP had more notable edges in a depression network, with thicker networks indicating more significant psychopathology (Moriarity, van Borkulo, & Alloy, 2021b). Concentration deficits and psychomotor problems (vs. other depression nodes) were the most influential in this study (Moriarity et al., 2021b). Moreover, higher CRP showed the largest associations with appetite changes and fatigue than other depression nodes in another large community sample (Moriarity, Horn, Kautz, Haslbeck, & Alloy, 2021a). Likewise, the polygenic risk score of CRP (but not IL-6 and other proinflammatory proteins) was most potently linked to fatigue and decreased anhedonia (Kappelmann et al., 2021). In addition, levels of triglyceride, total cholesterol, and insulin resistance, but not HDL, displayed the most substantial concurrent relations with higher depression severity in Korean adults (Nam, Peterson, Seo, Han, & Kang, 2021).

Therefore, the current study used CLPN to understand better the relations among surrogate lipid markers, proinflammatory proteins, and depression nodes across five time-points spanning nine years. This research aim is essential for multiple reasons. Globally, metabolic syndrome-linked disorders (e.g., diabetes) and depressive disorders have increased (Jeuring et al., 2018; Leon & Maddox, 2015). Enhancing knowledge of the modifiable risk factors and outcomes for depression and related immunometabolism problems can facilitate fine-tuning current evidence-based treatments (e.g., physical exercise-focused behavioral therapies; Li et al., 2017). Also, most studies examining the links among depression components, proinflammatory proteins, and surrogate lipid markers have been cross-sectional (e.g., Persons & Fiedorowicz, 2016), hindering weak causal inferences (Blanchard, Contreras, Kalkan, & Heeren, 2022). Thus, based on theory and evidence, we tested two hypotheses. First, we hypothesized that the within-person temporal (lag-1) network would show evidence more consistent with vulnerability models than scar theories (Hypothesis 1). Second, we expected that within and between persons, somatic symptoms (vs. depressed mood and interpersonal problems) would have stronger associations with levels of proinflammatory proteins (vs. surrogate lipid markers) (Hypothesis 2).

# Method

## Participants

The present study was a secondary analysis of merged open-access datasets from the Study of Women’s Health Across the Nation (SWAN) project (Greendale et al., 2010). At Wave 1 (W1), the all-female participants (*n* = 2,224) had a mean age of 45.96 years (*SD* = 2.67, range = 42–53) (refer to Table 1 in the manuscript). Table S1(a) in the online supplementary materials (OSM) details the descriptive statistics of demographic and study variables with the non-imputed dataset. Table S1(b) offers descriptive statistics on related variables not included in the final analyses.

## Procedures

Participants completed a depression self-report and biomarker data collection protocols at W1 (1997-1998), Wave 2 (W2; 1998-2000), Wave 3 (W3; 2000-2002), Wave 4 (W4; 2002-2004), and Wave 5 (W5; 2004-2006). These five time-points were selected as they contained data relevant to our research question. Both self-reports and biomarker assays were collected on the same day of the study visit (El Khoudary et al., 2016; McClure et al., 2014).

## Measures

**Surrogate lipid markers.** EDTA-treated plasma and enzymatic approaches determined the levels of triglycerides and LDL (Myers, Cooper, Winn, & Smith, 1989). Heparin-2M manganese chloride facilitated the extraction of HDL levels (Warnick & Albers, 1978). The radioimmunoassay (RIA) (DPC Coat-a-count, Los Angeles, California) method assessed serum insulin levels with monthly quality assurance checks (Diabetes Diagnostic Laboratory, University of Missouri, Columbia, Missouri). Also, the Hitachi 747-200 (Boehringer Mannheim Diagnostics, Indianapolis, Indiana) with the hexokinase-coupled reaction feature enabled the measurement of fasting glucose levels (Kelley-Hedgepeth et al., 2008).

**Proinflammatory proteins.** A clot-based turbidometric identification system assessed the fibrinogen level in frozen plasma preserved with citric acid (Medical Laboratory Automation Inc., Mt. Vernon, NY) (Falconi, Gold, & Janssen, 2016). The CRP level was determined by an ultrasensitive rate immunonephelometry approach with a lower identification limit (0.3 mg/L) (BN100; Dade-Behring, Marburg, Germany).

**Depression components.** Past-week depression components were measured with the Center for Epidemiologic Studies Depression (CES-D) scale (Radloff, 1977). Participants rated items on a 4-point Likert scale (0 = *rarely* to 3 = *most or all of the time*). We focused on three theory-based components derived from a recent factor analytic study in community adults: depressed mood; interpersonal problems; and somatic symptoms (Cosco et al., 2017).

## Statistical Analysis

All data analyses were conducted using *R* version 4.1.0 and *RStudio* version 1.4.1717 (*R* Core Team, 2021). Nodes represented components of depression (interpersonal problems, depressed mood, somatic symptoms), proinflammatory proteins and surrogate lipid markers (CRP, fibrinogen, HDL, fasting glucose, insulin, LDL, triglycerides), and covariates (age, estradiol (pg/mL), follicle-stimulating hormone (FSH) (mIU/mL), menopausal status (coded as 1 = premenopausal, 2 = early perimenopausal, 3 = late perimenopausal, 4 = post-menopausal)) (El Khoudary et al., 2016b; Persons et al., 2016). Table 1 shows the descriptive statistics of each node at distinct time-points with the multiply imputed dataset (cf. Table S1 for descriptive statistics with original dataset). Before network estimation, scores for all nodes were rescaled to range from 1 to 4 (matching the CES-D) to minimize biases due to variability differences (Fried et al., 2018). No outliers were identified (i.e., all skewness and kurtosis values were within normal limits).

Next, we used the panel data-graphical vector autoregressive (panelgvar) model (Epskamp, 2020) to determine three networks: (a) within-person temporal (lag-1) network (directed partial associations for the mean within-person effects across time); (b) within-person contemporaneous network (partial associations for the mean within-person effects within a time-point over and above temporal effects); (c) between-person network (partial associations for stable trait-level differences across time). We fit a non-regularized (unpruned) panelgvar model (Speyer et al., 2022). Model fit was evaluated with these fit statistics: confirmatory fit index (CFI; CFI ≥ .90), Tucker-Lewis Index (TLI; TLI ≥ .90), and root mean square error of approximation (RMSEA; RMSEA ≤ .060) (Hu & Bentler, 1999).

As our sample size was large (*n* = 2,224), we used an unpruned or non-regularized (vs. regularized) Gaussian graphical model to interpret network structures because it raises the chances of selecting the true model (Isvoranu & Epskamp, 2021). Non-regularized networks were fit using the *qgraph* (Epskamp, Borsboom, & Fried, 2018; Epskamp, Cramer, Waldorp, Schmittmann, & Borsboom, 2012) and *psychonetrics* (Epskamp, 2020) *R* packages. We uploaded analytic data syntax to OSF (<https://osf.io/upkyr/>). The non-regularized graphical least absolute shrinkage and selection operator (graphical LASSO) was used to estimate the structure of 100 regularized network models from sparse to dense (Epskamp, Kruis, & Marsman, 2017; Moriarity et al., 2021a; Williams & Rast, 2020).

To determine the accuracy of network edges, we computed the 95% confidence intervals (CI) of the edge weights with 1,000 bootstrap samples (Costenbader & Valente, 2003). Furthermore, only statistically significant edges (*p* < .001) and edges included ≥ 50% of the time across 1,000 bootstrap samples were regarded as stable (Betz et al., 2020; Epskamp, 2020). Cohen’s *d* effect sizes were calculated to ease interpretation (Dunlap, Cortina, Vaslow, & Burke, 1996; Rosenthal, 1994). Based on the literature (Mac Giollabhui et al., 2021), *d* ≥ 0.100 was interpreted as meaningful. We rendered edges that were accurate, stable, and with *d* ≥ 0.100 as significant. In addition, the Fruchterman–Reingold algorithm was used to organize the networks by locating the largest associations in the center and weaker associations toward the boundary and placing nodes with stronger relations closer to each other (Fruchterman & Reingold, 1991). Line thickness indicates the strength of association. Although bold blue lines signal positive relations, red dotted lines reflect negative ones. To test H1 formally, we used robust variance estimation (RVE) (Tanner-Smith, Tipton, & Polanin, 2016) to determine if substantial effect sizes consistent with vulnerability (vs. scar) theories were statistically significantly different. To evaluate H2, we utilized RVE to test the existence of significant effect size differences between substantial edges that included somatic symptoms, proinflammatory proteins, and their interaction.^[[1]](#footnote-2)^

# Results

## CLPN Model Fit Evaluation

The non-regularized CLPN model had good fit (CFI = 1.00, TLI = .95, RMSEA = .000, 90% CI [.000, .000]).

**Accuracy and** Stability of Networks

Figures S2(a)-(c) present the 95% CI plot that indicates the accuracy of all edges for the within-person temporal network, within-person contemporaneous network, and between-person network, respectively. The percentages of 95% CI of edges that did not cross the 0 value were 95.8% (182/190) for the temporal network, 98.9% (90/91) for the contemporaneous network, and 96.7% (88/91) for the between-person network. Tables S2(a)-(c) show the partial correlation statistics of each network, and Table S3(a)-(c) shows the frequency that each edge was included across all 1,000 bootstrap samples. The frequency of edges included in ≥ 50% of all bootstrap samples was 129 out of 196 edges (65.8%) for the temporal network, 54 out of 91 edges (59.3%) for the contemporaneous network, and 60 out of 91 edges (65.9%) for the between-person network. Thus, all networks showed a good degree of accuracy and stability.

## Within-Person Temporal (Lag-1) Network

Table 2 shows the parameter estimates for the within-person temporal (lag-1) network edges across distinct depression and surrogate immunometabolism constructs. Figure 1 displays all estimated fixed-effect within-person network standardized partial correlations. Table S4 displays parameter estimates of all lag-1 directed network edges within and across constructs.

**Scar Theories.** Within persons, depressed mood did not stably predict other immunometabolism markers at the next time-point. However, within-person increased somatic symptoms significantly predicted future increased fibrinogen (*d* = 0.188) (*p* < .001) rather than other lipid markers and proinflammatory proteins. Also, heightened interpersonal problems significantly predicted future higher fibrinogen (*d* = 0.129) and LDL (*d* = 0.331) (all *ps* < .001) instead of other lipid markers and proinflammatory proteins.

**Vulnerability Models.** These two surrogate immunometabolism markers significantly predicted all future depression nodes: (a) CRP (higher CRP 🡪 greater depressed mood: *d* = 1.072; higher CRP 🡪 greater somatic symptoms: *d* = 1.812; higher CRP 🡪 greater interpersonal problems: *d* = 2.112) (all *ps* < .001); and (b) HDL (higher HDL 🡪 stronger depressed mood: *d* = 0.196; higher HDL 🡪 stronger somatic symptoms: *d* = 0.162; higher HDL 🡪 stronger interpersonal problems: *d* = 0.134) (all *ps* < .001). Also, higher depressed mood was significantly predicted by previous higher LDL levels (*d* = 0.251, *p* < .001) instead of fibrinogen, glucose, insulin, and triglycerides. Greater somatic symptoms were significantly predicted by prior higher levels of fibrinogen (*d* = 0.156) and triglycerides (*d* = 0.174) (all *ps* < .001), but not fasting glucose, insulin, and LDL. More interpersonal problems were significantly predicted by prior higher LDL (*d* = 0.436, *p* < .001), but not fibrinogen, insulin, fasting glucose, and triglycerides.

The effect sizes from scar and vulnerability models did not significantly differ from one another (β = 0.081, 95% CI [-0.207, 0.368]). Thus, the findings did not support H1.

## Within-Person Contemporaneous Network

Figure 2 and Table S5 show all contemporaneous network edges parameter estimates and statistics after adjusting for within-person temporal relations and between-person differences. Within persons, greater depressed mood was significantly related to higher fasting glucose (*d* = 0.298, *p* < .001), but not CRP, fibrinogen, insulin, triglycerides, HDL, and LDL. Also, greater somatic symptoms were significantly associated with higher fasting glucose (*d* = 3.586, *p* < .001), but not fibrinogen, CRP, insulin, triglycerides, LDL, and HDL. Additionally, within-person greater interpersonal problems were significantly correlated with higher fibrinogen (*d* = 1.029), fasting glucose (*d* = 1.055) and HDL (*d* = 0.181) (all *ps* < .001), but not CRP, insulin, triglycerides, and LDL levels.

## Between-Person Network

Figure 3 and Table S6 show that between persons, stronger depressed mood was significantly related to higher CRP (*d* = 0.205) and fasting glucose (*d* = 0.138) (all *ps* < .001), but not fibrinogen, triglycerides, insulin, LDL, and HDL levels. Between persons, stronger somatic symptoms were significantly associated with higher CRP (*d* = 0.240), fasting glucose (*d* = 0.447), insulin (*d* = 0.231) (all *ps* < .001), but not fibrinogen, triglycerides, HDL, and LDL. Also, interpersonal problems were not stably related to any immunometabolism markers between persons. Inconsistent with H2, the strength of associations did not differ between significant edges with somatic symptoms (vs. depressed mood and interpersonal problems) (β = -0.003, 95% CI [-0.130, 0.125]), proinflammatory proteins (vs. proxy lipids) (β = 0.0144, 95% CI [-0.128, 0.157]), and their interaction (β = 0.007, 95% CI [-0.261, 0.274]).

# Discussion

Contrary to our hypotheses, findings provide consistent evidence for vulnerability models and scar theories, with small-to-large effect sizes. Further, somatic symptoms, depressed mood, and interpersonal problems had similarly strong positive relations with proinflammatory proteins and proxy lipid markers. We offer potential theoretical accounts on this topic based on outcomes produced by the current study's largely data-driven, cutting-edge CLPN. The within-person temporal network, rather than within-person contemporaneous and between-person networks, takes precedence when interpreting results because it provides directionality information.

Some notable temporal network relations emerged between components of depression and proinflammatory proteins. First, replicating and extending a recent meta-analysis with similar findings (Mac Giollabhui et al., 2021), higher CRP unidirectionally predicted later increased depressed mood, somatic symptoms, and interpersonal problems, but not vice versa. We observed large effect sizes of CRP (vs. other proxy immunometabolism markers) predicting depression components in the within-person temporal networks (*d* = 1.072 to 2.112). The unique biological properties of CRP (e.g., cardiovascular risk-enhancing attributes, increased fat storage) might contribute to those large effects, as evidenced by Mendelian randomization genetic (e.g., Khandaker et al., 2020) and related studies (Castanon, Lasselin, & Capuron, 2014) with hundreds of thousands of participants. Prognostically, suboptimal CRP and associated markers (e.g., fibrinogen, HDL, triglycerides, LDL levels) are probably proinflammatory proteins and surrogate lipid markers driving the etiology of depression. Thus, it is possible that modifying these proxy immunometabolism markers might efficiently treat depression and improve immunometabolism profiles. Also, within persons, contemporaneous networks revealed large positive cross-sectional effect sizes between somatic symptoms and glucose as well as interpersonal problems and glucose and fibrinogen (*d* = 1.029 to 3.589) above and beyond temporal effects. Such outliers suggest that the distinctive depression-associated mechanisms of excessive fibrinogen (e.g., increased arterial plaques and clots) and glucose (e.g., metabolism-altering characteristics) merit attention (Kucukgoncu et al., 2019; Von Känel, Bellingrath, & Kudielka, 2009).

Another notable observation was that there were larger effect sizes at the within- (vs. between-) person level (i.e., average significant *d* = 0.731 vs. 0.252). Such findings suggest that biological psychiatry can profit from conducting more studies with within-subject designs that capture person-specific fluctuations since effect size magnitudes can vary at the individual differences and within-person levels (Renna et al., 2020). Although longitudinal between-person analyses allow an inference that immunometabolism at a time-point predicts later depression across a sample, such group-level patterns might not extend to individuals across time (Wright & Woods, 2020).

Additionally, fibrinogen had a positive and small reciprocal effect on somatic symptoms over time. Such a result extends evidence for fibrinogen levels positively predicting depression indices (e.g., major depressive disorder) (Zainal & Newman, 2021b). Our findings support the idea that inflammatory processes tend to be more pronounced in atypical (vs. melancholic/mood-focused) depression characterized by bodily symptoms (Penninx, 2017). They also buttress the ‘sickness behavior’ hypothesis that somatic symptoms (e.g., psychomotor slowing, restless sleep) substantially predict increased proinflammatory proteins (Iob, Kirschbaum, & Steptoe, 2020).

Overall, our results highlight the importance of clarifying *unique* depression components that specific proinflammatory proteins positively impact. Plausibly, increased CRP and fibrinogen predicted heightened depression components, particularly somatic symptoms, by producing more proinflammatory cytokines from peripheral blood mononuclear cells (e.g., IL-6, tumor necrosis factor-α) (Haroon, Raison, & Miller, 2012). Proinflammatory cytokines might trigger and increase the enzyme indolamine-2,3-dioxygenase, which depletes monoamine precursors (i.e., antecedents of serotonin and dopamine such as tryptophan) by breaking it down into kynurenine (Felger, 2018). Eventually, reduced serotonin, dopamine, and norepinephrine synthesis and modified apoptosis and oxidative stress (Lamers et al., 2020) could contribute to elevated depression. Future basic science research should evaluate these notions.

Notably, temporal networks showed that excessive HDL predicted all depression components measured herein but not conversely. Further, temporal networks revealed positive feedback loops between LDL and depressed mood and LDL and interpersonal problems, but not LDL and somatic symptoms. Also, elevated triglycerides preceded more somatic symptoms (vs. other depression nodes) than vice versa, suggesting that this is an event that could occur in both community-dwelling adult women and men (Xu et al., 2021). Such observations agree that reducing hypertriglyceridemia is essential to treat and prevent the onset or recurrence of physical aspects of depression (Hamer, Batty, & Kivimaki, 2012). The state-of-the-art network analysis thus offers much information on the direction, magnitude, and possible reciprocal influence(s) among components of depression and surrogate lipid markers. Our results expand on cross-sectional meta-analytic evidence that HDL positively correlated with depression only among women (Shin, Suls, & Martin, 2008) and network analytic evidence that heightened HDL (vs. LDL and total cholesterol) coincided with more depressed mood (Jia et al., 2020). They also add to accruing evidence for the role of proxy markers of metabolic syndrome and poor glycemic control serving as risk factors for elevated depression in community adults (Mezuk, Eaton, Albrecht, & Golden, 2008; Watson et al., 2021).

Suboptimal levels of unique lipid markers heightened the risk of experiencing more distinct aspects of depression later, likely by dysregulating the HPA axis via excessive or blunted (vs. optimal) cortisol production (Mansur, Brietzke, & McIntyre, 2015). Other tenable mechanisms include decreased neurogenesis in reward- and executive functioning- related brain regions and connectivity between physiological states and synaptic plasticity (Goldsmith et al., 2020; Hamer et al., 2019; Zainal & Newman, 2021a). Plausibly, these processes can unfold with and without chronic social stressors and relate to somatic aspects (e.g., appetite changes, fatigue) of depression that often co-occur with motivational deficits and social withdrawal (Coccurello, 2019). Future prospective network analyses should examine these ideas.

Partially consistent with scar theories, somatic symptoms and interpersonal issues, but not depressed mood, preceded higher fibrinogen levels. More interpersonal problems, but not depressed mood and somatic symptoms, also forecasted increased LDL. Results extend evidence that more daily positive interpersonal events dovetailed with future reduced CRP and fibrinogen among women but not men (Sin, Graham-Engeland, & Almeida, 2015). They also build on evidence that rises in HDL or LDL levels (indicators of the buildup of fatty plaques in heart arteries) predicted depression in community adult women instead of men (Beydoun et al., 2015) and more cardiovascular events and rapid cognitive decline (Hua, Ma, Li, Zhong, & Xie, 2021). Most importantly, findings suggest improving lifestyle patterns to lessen depression and prevent dyslipidemia and heightened inflammation.

Study limitations merit attention. First, the all-female sample precluded the generalization of findings to the general population. Future studies should examine how sex assigned at birth might influence our CLPN-derived results due to documented sex differences in proinflammatory proteins, lipid markers, problem- versus emotion-focused coping approaches, and their interactions (Shimanoe et al., 2018). For example, sex could moderate within-person CRP-depression associations (Das, 2020) and relate to the hypothalamic-pituitary-gonadal axis, corticotropic-releasing hormone, cell death programming, and mitochondrial differences (Dantzer et al., 2021). Whereas women usually consume fatty acids in most cell metabolism processes, men mainly use amino acids and proteins (Demarest & McCarthy, 2015). For these and related biopsychosocial reasons, heightened depression occurs in more women than men (Shimamoto & Rappeneau, 2017), necessitating the recruitment of both genders in future studies. Second, as the current study was a secondary analysis, we were limited to available data. Other related chronic low-grade systemic proinflammatory proteins (e.g., IL-6), endocrine markers, and psychopathology components might have contributed to the current pattern of results. For example, IL-6 is instrumental in CRP and fibrinogen production, and inhibiting IL-6 with monoclonal antibodies affects lipid markers (Raison, Knight, & Pariante, 2018). Also, although controlling for age did not affect the results in this middle-aged sample, network associations might be more potent in middle-aged compared to younger adult women (Walker et al., 2021). Nonetheless, study strengths include the large sample size and the cutting-edge CLPN that separated within- and between-person relations and offered more information than traditional statistics. Moreover, our analyses adjusted for age, estradiol, follicle-stimulating hormone, and menopausal status.

Cognitive-behavioral therapies (CBT) that raise the consumption of foods with high soluble dietary fiber (e.g., oat bran, rye bran), reduce intake of sugary or low fiber foods and promote regular physical activity may facilitate those aims (Johansson-Persson et al., 2014; Li et al., 2017). Also, clinical science can profit from testing the efficacy of encouraging the consumption of a Mediterranean diet (e.g., olive oil, fish, fruits, vegetable) (Abenavoli et al., 2018) and improving sleep using evidence-based CBT strategies (Irwin et al., 2014). Further, findings highlight how optimizing immunometabolism profiles require enhancing social support (e.g., reducing loneliness), social engagement, and related contextual variables (cf. interpersonal theories; Walker, Ploubidis, & Fancourt, 2019; Wiebe, Helgeson, & Berg, 2016). Mounting evidence indicates that these CBT approaches could alleviate depression and enhance immunometabolism profiles long-term (Shomaker et al., 2017), which merits more attention.

References

Abenavoli, L., Di Renzo, L., Boccuto, L., Alwardat, N., Gratteri, S., & De Lorenzo, A. (2018). Health benefits of Mediterranean diet in nonalcoholic fatty liver disease. *Expert Review of Gastroenterology & Hepatology, 12*, 873-881. doi:10.1080/17474124.2018.1503947

Betz, L. T., Penzel, N., Kambeitz-Ilankovic, L., Rosen, M., Chisholm, K., Stainton, A., . . . consortium, P. (2020). General psychopathology links burden of recent life events and psychotic symptoms in a network approach. *npj Schizophrenia, 6*, 40. doi:10.1038/s41537-020-00129-w

Beydoun, M. A., Beydoun, H. A., Dore, G. A., Fanelli-Kuczmarski, M. T., Evans, M. K., & Zonderman, A. B. (2015). Total serum cholesterol, atherogenic indices and their longitudinal association with depressive symptoms among US adults. *Translational Psychiatry, 5*, e518. doi:10.1038/tp.2015.4

Blanchard, M. A., Contreras, A., Kalkan, R. B., & Heeren, A. (2022). Auditing the research practices and statistical analyses of the group-level temporal network approach to psychological constructs: A systematic scoping review. *Behavior Research Methods*. doi:10.3758/s13428-022-01839-y

Borsboom, D., Deserno, M. K., Rhemtulla, M., Epskamp, S., Fried, E. I., McNally, R. J., . . . Waldorp, L. J. (2021). Network analysis of multivariate data in psychological science. *Nature Reviews Methods Primers, 1*, 1-18. doi:10.1038/s43586-021-00055-w

Castanon, N., Lasselin, J., & Capuron, L. (2014). Neuropsychiatric comorbidity in obesity: Role of inflammatory processes. *Frontiers in Endocrinology, 5*, 74. doi:10.3389/fendo.2014.00074

Cecconello, C., Clària Ribas, P., & Norling, L. V. (2022). Resolving acute inflammation; what happens when inflammation goes haywire? How can it get back in line? *Diet, Inflammation, and Health* (pp. 113-162). doi: 10.1016/b978-0-12-822130-3.00018-1

Chen, S., Zhang, Q., Dai, G., Hu, J., Zhu, C., Su, L., & Wu, X. (2016). Association of depression with pre-diabetes, undiagnosed diabetes, and previously diagnosed diabetes: A meta-analysis. *Endocrine, 53*, 35-46. doi:10.1007/s12020-016-0869-x

Coccurello, R. (2019). Anhedonia in depression symptomatology: Appetite dysregulation and defective brain reward processing. *Behavioural Brain Research, 372*, 112041. doi:10.1016/j.bbr.2019.112041

Colasanto, M., Madigan, S., & Korczak, D. J. (2020). Depression and inflammation among children and adolescents: A meta-analysis. *Journal of Affective Disorders, 277*, 940-948. doi:10.1016/j.jad.2020.09.025

Cosco, T. D., Prina, M., Stubbs, B., & Wu, Y. T. (2017). Reliability and validity of the Center for Epidemiologic Studies Depression Scale in a population-based cohort of middle-aged U.S. adults. *Journal of Nursing Measurement, 25*, 476-485. doi:10.1891/1061-3749.25.3.476

Costenbader, E., & Valente, T. W. (2003). The stability of centrality measures when networks are sampled. *Social Networks, 25*, 283-307. doi:10.1016/s0378-8733(03)00012-1

Dantzer, R., Casaril, A., & Vichaya, E. (2021). Inflammation and depression: Is immunometabolism the missing link? In M. Berk, M. Leboyer & I. E. Sommer (Eds.), *Immuno-Psychiatry: Facts and Prospects* (pp. 259-287). Cham: Springer International Publishing. doi: 10.1007/978-3-030-71229-7_16

Dantzer, R., O'Connor, J. C., Freund, G. G., Johnson, R. W., & Kelley, K. W. (2008). From inflammation to sickness and depression: When the immune system subjugates the brain. *Nature reviews. Neuroscience, 9*, 46-56. doi:10.1038/nrn2297

Das, A. (2020). Chronic ongoing stressors and C-reactive protein: A within-person study. *Journal of Aging and Health, 32*, 892-903. doi:10.1177/0898264319862419

Dedoncker, J., Vanderhasselt, M. A., Ottaviani, C., & Slavich, G. M. (2021). Mental health during the COVID-19 pandemic and beyond: The importance of the vagus nerve for biopsychosocial resilience. *Neuroscience & Biobehavioral Reviews, 125*, 1-10. doi:10.1016/j.neubiorev.2021.02.010

Demarest, T. G., & McCarthy, M. M. (2015). Sex differences in mitochondrial (dys)function: Implications for neuroprotection. *Journal of Bioenergetics and Biomembranes, 47*, 173-188. doi:10.1007/s10863-014-9583-7

Dias, J. P., Joseph, J. J., Kluwe, B., Zhao, S., Shardell, M., Seeman, T., . . . Golden, S. H. (2020). The longitudinal association of changes in diurnal cortisol features with fasting glucose: MESA. *Psychoneuroendocrinology, 119*, 104698. doi:10.1016/j.psyneuen.2020.104698

Duivis, H. E., de Jonge, P., Penninx, B. W., Na, B. Y., Cohen, B. E., & Whooley, M. A. (2011). Depressive symptoms, health behaviors, and subsequent inflammation in patients with coronary heart disease: prospective findings from the heart and soul study. *American Journal of Psychiatry, 168*, 913-920. doi:10.1176/appi.ajp.2011.10081163

Dunlap, W. P., Cortina, J. M., Vaslow, J. B., & Burke, M. J. (1996). Meta-analysis of experiments with matched groups or repeated measures designs. *Psychological Methods, 1*, 170-177. doi:10.1037/1082-989x.1.2.170

El Khoudary, S. R., Hutchins, P. M., Matthews, K. A., Brooks, M. M., Orchard, T. J., Ronsein, G. E., & Heinecke, J. W. (2016). Cholesterol efflux capacity and subclasses of HDL particles in healthy women transitioning through menopause. *Journal of Clinical Endocrinology and Metabolism, 101*, 3419-3428. doi:10.1210/jc.2016-2144

Ellins, E. A., Rees, D. A., Deanfield, J. E., Steptoe, A., & Halcox, J. P. (2017). Increased fibrinogen responses to psychophysiological stress predict future endothelial dysfunction implications for cardiovascular disease? *Brain, Behavior, and Immunity, 60*, 233-239. doi:10.1016/j.bbi.2016.10.017

Epskamp, S. (2020). Psychometric network models from time-series and panel data. *Psychometrika, 85*, 206-231. doi:10.1007/s11336-020-09697-3

Epskamp, S., Borsboom, D., & Fried, E. I. (2018). Estimating psychological networks and their accuracy: A tutorial paper. *Behavior Research Methods, 50*, 195-212. doi:10.3758/s13428-017-0862-1

Epskamp, S., Cramer, A. O., Waldorp, L. J., Schmittmann, V. D., & Borsboom, D. (2012). qgraph: Network visualizations of relationships in psychometric data. *Journal of Statistical Software, 48*, 1-18. doi:10.18637/jss.v048.i04

Epskamp, S., Kruis, J., & Marsman, M. (2017). Estimating psychopathological networks: Be careful what you wish for. *PLoS One, 12*, e0179891. doi:10.1371/journal.pone.0179891

Falconi, A. M., Gold, E. B., & Janssen, I. (2016). The longitudinal relation of stress during the menopausal transition to fibrinogen concentrations: Results from the study of women's health across the nation. *Menopause, 23*, 518-527. doi:10.1097/GME.0000000000000579

Felger, J. C. (2018). Imaging the role of inflammation in mood and anxiety-related disorders. *Current Neuropharmacology, 16*, 533-558. doi:10.2174/1570159X15666171123201142

Felger, J. C., Haroon, E., Patel, T. A., Goldsmith, D. R., Wommack, E. C., Woolwine, B. J., . . . Miller, A. H. (2020). What does plasma CRP tell us about peripheral and central inflammation in depression? *Molecular Psychiatry, 25*, 1301-1311. doi:10.1038/s41380-018-0096-3

Felger, J. C., Li, Z., Haroon, E., Woolwine, B. J., Jung, M. Y., Hu, X., & Miller, A. H. (2016). Inflammation is associated with decreased functional connectivity within corticostriatal reward circuitry in depression. *Molecular Psychiatry, 21*, 1358-1365.

Feng, X., & Astell-Burt, T. (2017). Impact of a type 2 diabetes diagnosis on mental health, quality of life, and social contacts: A longitudinal study. *BMJ Open Diabetes Research and Care, 5*, e000198. doi:10.1136/bmjdrc-2016-000198

Fried, E. I., Eidhof, M. B., Palic, S., Costantini, G., Huisman-van Dijk, H. M., Bockting, C. L. H., . . . Karstoft, K. I. (2018). Replicability and generalizability of posttraumatic stress disorder (PTSD) networks: A cross-cultural multisite study of PTSD symptoms in four trauma patient samples. *Clinical Psychological Science, 6*, 335-351. doi:10.1177/2167702617745092

Fried, E. I., von Stockert, S., Haslbeck, J. M. B., Lamers, F., Schoevers, R. A., & Penninx, B. W. J. H. (2020). Using network analysis to examine links between individual depressive symptoms, inflammatory markers, and covariates. *Psychological Medicine, 50*, 2682-2690. doi:10.1017/S0033291719002770

Fruchterman, T. M. J., & Reingold, E. M. (1991). Graph drawing by force-directed placement. *Software: Practice and Experience, 21*, 1129-1164. doi:10.1002/spe.4380211102

Goldsmith, D. R., Bekhbat, M., Le, N. A., Chen, X., Woolwine, B. J., Li, Z., . . . Felger, J. C. (2020). Protein and gene markers of metabolic dysfunction and inflammation together associate with functional connectivity in reward and motor circuits in depression. *Brain, Behavior, and Immunity, 88*, 193-202. doi:10.1016/j.bbi.2020.05.013

Gouin, J. P., Wrosch, C., McGrath, J., & Booij, L. (2020). Interpersonal capitalization moderates the associations of chronic caregiving stress and depression with inflammation. *Psychoneuroendocrinology, 112*, 104509. doi:10.1016/j.psyneuen.2019.104509

Greendale, G. A., Wight, R. G., Huang, M. H., Avis, N., Gold, E. B., Joffe, H., . . . Karlamangla, A. S. (2010). Menopause-associated symptoms and cognitive performance: Results from the study of women's health across the nation. *American Journal of Epidemiology, 171*, 1214-1224. doi:10.1093/aje/kwq067

Hamer, J. A., Testani, D., Mansur, R. B., Lee, Y., Subramaniapillai, M., & McIntyre, R. S. (2019). Brain insulin resistance: A treatment target for cognitive impairment and anhedonia in depression. *Experimental Neurology, 315*, 1-8. doi:10.1016/j.expneurol.2019.01.016

Hamer, M., Batty, G. D., & Kivimaki, M. (2012). Risk of future depression in people who are obese but metabolically healthy: The English longitudinal study of ageing. *Molecular Psychiatry, 17*, 940-945. doi:10.1038/mp.2012.30

Haroon, E., Raison, C. L., & Miller, A. H. (2012). Psychoneuroimmunology meets neuropsychopharmacology: Translational implications of the impact of inflammation on behavior. *Neuropsychopharmacology, 37*, 137-162. doi:10.1038/npp.2011.205

Hiles, S. A., Revesz, D., Lamers, F., Giltay, E., & Penninx, B. W. (2016). Bidirectional prospective associations of metabolic syndrome components with depression, anxiety, and antidepressant use. *Depression and Anxiety, 33*, 754-764. doi:10.1002/da.22512

Hu, L. t., & Bentler, P. M. (1999). Cutoff criteria for fit indexes in covariance structure analysis: Conventional criteria versus new alternatives. *Structural Equation Modeling: A Multidisciplinary Journal, 6*, 1-55. doi:10.1080/10705519909540118

Hua, R., Ma, Y., Li, C., Zhong, B., & Xie, W. (2021). Low levels of low-density lipoprotein cholesterol and cognitive decline. *Science Bulletin, 66*, 1684-1690. doi:10.1016/j.scib.2021.02.018

Ignacio, Z. M., da Silva, R. S., Plissari, M. E., Quevedo, J., & Reus, G. Z. (2019). Physical exercise and neuroinflammation in major depressive disorder. *Molecular Neurobiology, 56*, 8323-8335. doi:10.1007/s12035-019-01670-1

Iob, E., Kirschbaum, C., & Steptoe, A. (2020). Persistent depressive symptoms, HPA-axis hyperactivity, and inflammation: The role of cognitive-affective and somatic symptoms. *Molecular Psychiatry, 25*, 1130-1140. doi:10.1038/s41380-019-0501-6

Irwin, M. R., Olmstead, R., Carrillo, C., Sadeghi, N., Breen, E. C., Witarama, T., . . . Nicassio, P. (2014). Cognitive behavioral therapy vs. Tai Chi for late life insomnia and inflammatory risk: A randomized controlled comparative efficacy trial. *Sleep, 37*, 1543-1552. doi:10.5665/sleep.4008

Isvoranu, A. M., & Epskamp, S. (2021). Which estimation method to choose in network psychometrics? Deriving guidelines for applied researchers. *Psychological Methods*. doi:10.1037/met0000439

Jeuring, H. W., Comijs, H. C., Deeg, D. J. H., Stek, M. L., Huisman, M., & Beekman, A. T. F. (2018). Secular trends in the prevalence of major and subthreshold depression among 55-64-year olds over 20 years. *Psychological Medicine, 48*, 1824-1834. doi:10.1017/S0033291717003324

Jia, Q. F., Yang, H. X., Zhuang, N. N., Yin, X. Y., Zhu, Z. H., Yuan, Y., . . . Hui, L. (2020). The role of lipoprotein profile in depression and cognitive performance: A network analysis. *Scientific Reports, 10*, 20704. doi:10.1038/s41598-020-77782-9

Johansson-Persson, A., Ulmius, M., Cloetens, L., Karhu, T., Herzig, K. H., & Onning, G. (2014). A high intake of dietary fiber influences C-reactive protein and fibrinogen, but not glucose and lipid metabolism, in mildly hypercholesterolemic subjects. *European Journal of Nutrition, 53*, 39-48. doi:10.1007/s00394-013-0496-8

Kappelmann, N., Czamara, D., Rost, N., Moser, S., Schmoll, V., Trastulla, L., . . . Arloth, J. (2021). Polygenic risk for immuno-metabolic markers and specific depressive symptoms: A multi-sample network analysis study. *Brain, Behavior, and Immunity, 95*, 256-268. doi:10.1016/j.bbi.2021.03.024

Kelley-Hedgepeth, A., Lloyd-Jones, D. M., Colvin, A., Matthews, K. A., Johnston, J., Sowers, M. R., . . . Investigators, S. (2008). Ethnic differences in C-reactive protein concentrations. *Clinical Chemistry, 54*, 1027-1037. doi:10.1373/clinchem.2007.098996

Khandaker, G. M., Zuber, V., Rees, J. M. B., Carvalho, L., Mason, A. M., Foley, C. N., . . . Burgess, S. (2020). Shared mechanisms between coronary heart disease and depression: Findings from a large UK general population-based cohort. *Molecular Psychiatry, 25*, 1477-1486. doi:10.1038/s41380-019-0395-3

Konsman, J. P. (2019). Inflammation and depression: A nervous plea for psychiatry to not become immune to interpretation. *Pharmaceuticals, 12*. doi:10.3390/ph12010029

Kucukgoncu, S., Kosir, U., Zhou, E., Sullivan, E., Srihari, V. H., & Tek, C. (2019). Glucose metabolism dysregulation at the onset of mental illness is not limited to first episode psychosis: A systematic review and meta-analysis. *Early Intervention in Psychiatry, 13*, 1021-1031. doi:10.1111/eip.12749

Lafitte, M., Tastet, S., Perez, P., Serise, M. A., Grandoulier, A. S., Aouizerate, B., . . . Couffinhal, T. (2015). High sensitivity C reactive protein, fibrinogen levels and the onset of major depressive disorder in post-acute coronary syndrome. *BMC Cardiovascular Disorders, 15*, 23. doi:10.1186/s12872-015-0015-3

Lamers, F., Milaneschi, Y., Smit, J. H., Schoevers, R. A., Wittenberg, G., & Penninx, B. W. J. H. (2019). Longitudinal association between depression and inflammatory markers: Results from the Netherlands study of depression and anxiety. *Biological Psychiatry, 85*, 829-837. doi:10.1016/j.biopsych.2018.12.020

Lamers, F., Milaneschi, Y., Vinkers, C. H., Schoevers, R. A., Giltay, E. J., & Penninx, B. (2020). Depression profilers and immuno-metabolic dysregulation: Longitudinal results from the NESDA study. *Brain, Behavior, and Immunity, 88*, 174-183. doi:10.1016/j.bbi.2020.04.002

Lamers, F., Vogelzangs, N., Merikangas, K. R., de Jonge, P., Beekman, A. T., & Penninx, B. W. (2013). Evidence for a differential role of HPA-axis function, inflammation and metabolic syndrome in melancholic versus atypical depression. *Molecular Psychiatry, 18*, 692-699. doi:10.1038/mp.2012.144

Leon, B. M., & Maddox, T. M. (2015). Diabetes and cardiovascular disease: Epidemiology, biological mechanisms, treatment recommendations and future research. *World Journal of Diabetes, 6*, 1246-1258. doi:10.4239/wjd.v6.i13.1246

Li, C., Xu, D., Hu, M., Tan, Y., Zhang, P., Li, G., & Chen, L. (2017). A systematic review and meta-analysis of randomized controlled trials of cognitive behavior therapy for patients with diabetes and depression. *Journal of Psychosomatic Research, 95*, 44-54. doi:10.1016/j.jpsychores.2017.02.006

Mac Giollabhui, N., Ng, T. H., Ellman, L. M., & Alloy, L. B. (2021). The longitudinal associations of inflammatory biomarkers and depression revisited: Systematic review, meta-analysis, and meta-regression. *Molecular Psychiatry, 26*, 3302-3314. doi:10.1038/s41380-020-00867-4

Macleod, C. M., & Avery, O. T. (1941). The occurrence during acute infections of a protein not normally present in the blood: II. Isolation and properties of the reactive protein. *Journal of Experimental Medicine, 73*, 183-190. doi:10.1084/jem.73.2.183

Majd, M., Saunders, E. F. H., & Engeland, C. G. (2020). Inflammation and the dimensions of depression: A review. *Frontiers in Neuroendocrinology, 56*, 100800. doi:10.1016/j.yfrne.2019.100800

Mansur, R. B., Brietzke, E., & McIntyre, R. S. (2015). Is there a "metabolic-mood syndrome"? A review of the relationship between obesity and mood disorders. *Neuroscience & Biobehavioral Reviews, 52*, 89-104. doi:10.1016/j.neubiorev.2014.12.017

Marz, W., Kleber, M. E., Scharnagl, H., Speer, T., Zewinger, S., Ritsch, A., . . . Laufs, U. (2017). HDL cholesterol: Reappraisal of its clinical relevance. *Clinical Research in Cardiology, 106*, 663-675. doi:10.1007/s00392-017-1106-1

McClure, C. K., El Khoudary, S. R., Karvonen-Gutierrez, C. A., Ylitalo, K. R., Tomey, K., VoPham, T., . . . Harlow, S. (2014). Prospective associations between inflammatory and hemostatic markers and physical functioning limitations in mid-life women: Longitudinal results of the Study of Women's Health Across the Nation (SWAN). *Experimental Gerontology, 49*, 19-25. doi:10.1016/j.exger.2013.10.016

Mezuk, B., Eaton, W. W., Albrecht, S., & Golden, S. H. (2008). Depression and type 2 diabetes over the lifespan: A meta-analysis. *Diabetes Care, 31*, 2383-2390. doi:10.2337/dc08-0985

Michels, N., van Aart, C., Morisse, J., Mullee, A., & Huybrechts, I. (2021). Chronic inflammation towards cancer incidence: A systematic review and meta-analysis of epidemiological studies. *Critical Reviews in Oncology/Hematology, 157*, 103177. doi:10.1016/j.critrevonc.2020.103177

Moriarity, D. P., Horn, S. R., Kautz, M. M., Haslbeck, J. M. B., & Alloy, L. B. (2021a). How handling extreme C-reactive protein (CRP) values and regularization influences CRP and depression criteria associations in network analyses. *Brain, Behavior, and Immunity, 91*, 393-403. doi:10.1016/j.bbi.2020.10.020

Moriarity, D. P., van Borkulo, C., & Alloy, L. B. (2021b). Inflammatory phenotype of depression symptom structure: A network perspective. *Brain, Behavior, and Immunity, 93*, 35-42. doi:10.1016/j.bbi.2020.12.005

Myers, G. L., Cooper, G. R., Winn, C. L., & Smith, S. J. (1989). The Centers for Disease Control-National Heart, Lung and Blood Institute Lipid Standardization Program: An approach to accurate and precise lipid measurements. *Clinics in Laboratory Medicine, 9*, 105-136. doi:10.1016/s0272-2712(18)30645-0

Nam, S. M., Peterson, T. A., Seo, K. Y., Han, H. W., & Kang, J. I. (2021). Discovery of depression-associated factors from a nationwide population-based survey: Epidemiological study using machine learning and network analysis. *Journal of Medical Internet Research, 23*, e27344. doi:10.2196/27344

Pan, A., Keum, N., Okereke, O. I., Sun, Q., Kivimaki, M., Rubin, R. R., & Hu, F. B. (2012). Bidirectional association between depression and metabolic syndrome: A systematic review and meta-analysis of epidemiological studies. *Diabetes Care, 35*, 1171-1180. doi:10.2337/dc11-2055

Peirce, J. M., & Alvina, K. (2019). The role of inflammation and the gut microbiome in depression and anxiety. *Journal of Neuroscience Research, 97*, 1223-1241. doi:10.1002/jnr.24476

Penninx, B. W. (2017). Depression and cardiovascular disease: Epidemiological evidence on their linking mechanisms. *Neuroscience & Biobehavioral Reviews, 74*, 277-286. doi:10.1016/j.neubiorev.2016.07.003

Persons, J. E., & Fiedorowicz, J. G. (2016). Depression and serum low-density lipoprotein: A systematic review and meta-analysis. *Journal of Affective Disorders, 206*, 55-67. doi:10.1016/j.jad.2016.07.033

R Core Team. (2021). *R: A language and environment for statistical computing*. Vienna, Austria: R Foundation for Statistical Computing. URL: <https://www.R-project.org/>.

Radloff, L. S. (1977). The CES-D Scale: A self-report depression scale for research in the general population. *Applied Psychological Measurement, 1*, 385-401. doi:10.1177/014662167700100306

Raison, C. L., Knight, J. M., & Pariante, C. (2018). Interleukin (IL)-6: A good kid hanging out with bad friends (and why sauna is good for health). *Brain, Behavior, and Immunity, 73*, 1-2. doi:10.1016/j.bbi.2018.06.008

Renna, M. E., Shrout, M. R., Madison, A. A., Alfano, C. M., Povoski, S. P., Lipari, A. M., . . . Kiecolt-Glaser, J. K. (2020). Within-person changes in cancer-related distress predict breast cancer survivors' inflammation across treatment. *Psychoneuroendocrinology, 121*, 104866. doi:10.1016/j.psyneuen.2020.104866

Revicki, D. A., Travers, K., Wyrwich, K. W., Svedsater, H., Locklear, J., Mattera, M. S., . . . Montgomery, S. (2012). Humanistic and economic burden of generalized anxiety disorder in North America and Europe. *Journal of Affective Disorders, 140*, 103-112. doi:10.1016/j.jad.2011.11.014

Roefs, A., Fried, E. I., Kindt, M., Martijn, C., Elzinga, B., Evers, A. W. M., . . . Jansen, A. (2022). A new science of mental disorders: Using personalised, transdiagnostic, dynamical systems to understand, model, diagnose and treat psychopathology. *Behaviour Research and Therapy, 153*. doi:10.1016/j.brat.2022.104096

Rosenthal, R. (1994). Parametric measures of effect size. In H. Cooper & L. V. Hedges (Eds.), *The handbook of research synthesis*. New York, NY: Russell Sage Foundation.

Rotella, F., & Mannucci, E. (2013). Diabetes mellitus as a risk factor for depression. A meta-analysis of longitudinal studies. *Diabetes Research and Clinical Practice, 99*, 98-104. doi:10.1016/j.diabres.2012.11.022

Shimamoto, A., & Rappeneau, V. (2017). Sex-dependent mental illnesses and mitochondria. *Schizophrenia Research, 187*, 38-46. doi:10.1016/j.schres.2017.02.025

Shimanoe, C., Hara, M., Nishida, Y., Nanri, H., Otsuka, Y., Horita, M., . . . Tanaka, K. (2018). Coping strategy and social support modify the association between perceived stress and C-reactive protein: A longitudinal study of healthy men and women. *Stress, 21*, 237-246. doi:10.1080/10253890.2018.1435638

Shin, J. Y., Suls, J., & Martin, R. (2008). Are cholesterol and depression inversely related? A meta-analysis of the association between two cardiac risk factors. *Annals of Behavioral Medicine, 36*, 33-43. doi:10.1007/s12160-008-9045-8

Shomaker, L. B., Kelly, N. R., Radin, R. M., Cassidy, O. L., Shank, L. M., Brady, S. M., . . . Yanovski, J. A. (2017). Prevention of insulin resistance in adolescents at risk for type 2 diabetes with depressive symptoms: 1-year follow-up of a randomized trial. *Depression and Anxiety, 34*, 866-876. doi:10.1002/da.22617

Simpson, C. A., Diaz-Arteche, C., Eliby, D., Schwartz, O. S., Simmons, J. G., & Cowan, C. S. M. (2021). The gut microbiota in anxiety and depression - A systematic review. *Clinical Psychology Review, 83*, 101943. doi:10.1016/j.cpr.2020.101943

Sin, N. L., Graham-Engeland, J. E., & Almeida, D. M. (2015). Daily positive events and inflammation: Findings from the National Study of Daily Experiences. *Brain, Behavior, and Immunity, 43*, 130-138. doi:10.1016/j.bbi.2014.07.015

Sivertsen, H., Bjorklof, G. H., Engedal, K., Selbaek, G., & Helvik, A. S. (2015). Depression and quality of life in older persons: A review. *Dementia and Geriatric Cognitive Disorders, 40*, 311-339. doi:10.1159/000437299

Smith, K. J., Gavey, S., NE, R. I., Kontari, P., & Victor, C. (2020). The association between loneliness, social isolation and inflammation: A systematic review and meta-analysis. *Neuroscience and Biobehavioral Reviews, 112*, 519-541. doi:10.1016/j.neubiorev.2020.02.002

Speyer, L. G., Ushakova, A., Hall, H. A., Luciano, M., Auyeung, B., & Murray, A. L. (2022). Analyzing dynamic change in children's socioemotional development using the strengths and difficulties questionnaire in a large United Kingdom longitudinal study. *Journal of Psychopathology and Clinical Science, 131*, 162-171. doi:10.1037/abn0000714

Suvarna, B., Suvarna, A., Phillips, R., Juster, R. P., McDermott, B., & Sarnyai, Z. (2020). Health risk behaviours and allostatic load: A systematic review. *Neuroscience & Biobehavioral Reviews, 108*, 694-711. doi:10.1016/j.neubiorev.2019.12.020

Tanner-Smith, E. E., Tipton, E., & Polanin, J. R. (2016). Handling complex meta-analytic data structures using robust variance estimates: A tutorial in R. *Journal of Developmental and Life-Course Criminology, 2*, 85-112. doi:10.1007/s40865-016-0026-5

Thayer, J. F., & Fischer, J. E. (2009). Heart rate variability, overnight urinary norepinephrine and C-reactive protein: evidence for the cholinergic anti-inflammatory pathway in healthy human adults. *Journal of Internal Medicine, 265*, 439-447. doi:10.1111/j.1365-2796.2008.02023.x

Torres-Platas, S. G., Cruceanu, C., Chen, G. G., Turecki, G., & Mechawar, N. (2014). Evidence for increased microglial priming and macrophage recruitment in the dorsal anterior cingulate white matter of depressed suicides. *Brain, Behavior, and Immunity, 42*, 50-59. doi:10.1016/j.bbi.2014.05.007

Valkanova, V., Ebmeier, K. P., & Allan, C. L. (2013). CRP, IL-6 and depression: A systematic review and meta-analysis of longitudinal studies. *Journal of Affective Disorders, 150*, 736-744. doi:10.1016/j.jad.2013.06.004

Vingeliene, S., Hiyoshi, A., Lentjes, M., Fall, K., & Montgomery, S. (2019). Longitudinal analysis of loneliness and inflammation at older ages: English longitudinal study of ageing. *Psychoneuroendocrinology, 110*, 104421. doi:10.1016/j.psyneuen.2019.104421

Von Känel, R., Bellingrath, S., & Kudielka, B. M. (2009). Association between longitudinal changes in depressive symptoms and plasma fibrinogen levels in school teachers. *Psychophysiology, 46*, 473-480. doi:10.1111/j.1469-8986.2009.00788.x

Walker, E., Ploubidis, G., & Fancourt, D. (2019). Social engagement and loneliness are differentially associated with neuro-immune markers in older age: Time-varying associations from the English Longitudinal Study of Ageing. *Brain, Behavior, and Immunity, 82*, 224-229. doi:10.1016/j.bbi.2019.08.189

Walker, J. L., Slavish, D. C., Dolan, M., Dietch, J. R., Wardle-Pinkston, S., Messman, B., . . . Taylor, D. J. (2021). Age-dependent associations among insomnia, depression, and inflammation in nurses. *Psychology & Health, 36*, 967-984. doi:10.1080/08870446.2020.1805450

Warnick, G. R., & Albers, J. J. (1978). A comprehensive evaluation of the heparin–manganese precipitation procedure for estimating high density lipoprotein cholesterol. *Journal of Lipid Research, 19*, 65-76. doi:10.1016/s0022-2275(20)41577-9

Watson, K. T., Simard, J. F., Henderson, V. W., Nutkiewicz, L., Lamers, F., Nasca, C., . . . Penninx, B. (2021). Incident major depressive disorder predicted by three measures of insulin resistance: A Dutch cohort study. *American Journal of Psychiatry, 178*, 914-920. doi:10.1176/appi.ajp.2021.20101479

Wiebe, D. J., Helgeson, V., & Berg, C. A. (2016). The social context of managing diabetes across the life span. *American Psychologist, 71*, 526-538. doi:10.1037/a0040355

Williams, D. R., & Rast, P. (2020). Back to the basics: Rethinking partial correlation network methodology. *British Journal of Mathematical and Statistical Psychology, 73*, 187-212. doi:10.1111/bmsp.12173

Wright, A. G. C., & Woods, W. C. (2020). Personalized models of psychopathology. *Annual Review of Clinical Psychology, 16*, 49-74. doi:10.1146/annurev-clinpsy-102419-125032

Xu, L., Wang, K., Wang, S., Liu, L., Lv, X., & Song, Y. (2021). Sex differences in the association between serum lipids and depressive symptoms: A longitudinal population-based study. *Journal of Affective Disorders, 291*, 154-162. doi:10.1016/j.jad.2021.05.011

Zainal, N. H., & Newman, M. G. (2021a). Depression and worry symptoms predict future executive functioning impairment via inflammation. *Psychological Medicine*, 1-11. doi:10.1017/S0033291721000398

Zainal, N. H., & Newman, M. G. (2021b). Increased inflammation predicts nine-year change in major depressive disorder diagnostic status. *Journal of Abnormal Psychology, 130*, 829–840. doi:10.1037/abn0000716

Zainal, N. H., & Newman, M. G. (2021c). Larger increase in trait negative affect is associated with greater future cognitive decline and vice versa across 23 years. *Depression and Anxiety, 38*, 146–160. doi:10.1002/da.23093

Zainal, N. H., & Newman, M. G. (2022). Inflammation mediates depression and generalized anxiety symptoms predicting executive function impairment after 18 years. *Journal of Affective Disorders, 296*, 465-475. doi:10.1016/j.jad.2021.08.077

Zhang, S. F., Chen, H. M., Xiong, J. N., Liu, J., Xiong, J., Xie, J. Z., . . . Qu, N. (2022). Comparison of cognitive impairments with lipid profiles and inflammatory biomarkers in unipolar and bipolar depression. *Journal of Psychiatric Research, 150*, 300-306. doi:10.1016/j.jpsychires.2022.04.002

Table 1

*Descriptive Statistics of Network Nodes Across All Waves for Multiply Imputed Dataset*

|  | Wave 1 | | | Wave 2 | | | Wave 4 | | | Wave 6 | | | Wave 8 | | |
| --- | --- | --- | --- | --- | --- | --- | --- | --- | --- | --- | --- | --- | --- | --- | --- |
|  | *n* | *M/%* | (SD) | *n* | *M/%* | (SD) | *n* | *M/%* | (SD) | *n* | *M/%* | (SD) | *n* | *M/%* | (SD) |
| Age | 2224 | 1.524 | (0.627) | 2224 | 1.495 | (0.621) | 2224 | 1.473 | (0.600) | 2224 | 1.466 | (0.597) | 2224 | 1.429 | (0.588) |
| Age (unscaled) | 2224 | 45.964 | (2.674) | 2224 | 46.996 | (2.684) | 2224 | 49.031 | (2.694) | 2224 | 51.049 | (2.684) | 2224 | 53.094 | (2.689) |
| Depressed mood | 2224 | 1.799 | (0.612) | 2224 | 1.785 | (0.629) | 2224 | 1.768 | (0.618) | 2224 | 1.781 | (0.637) | 2224 | 1.699 | (0.584) |
| Somatic symptoms | 2224 | 1.426 | (0.621) | 2224 | 1.390 | (0.594) | 2224 | 1.398 | (0.599) | 2224 | 1.365 | (0.58) | 2224 | 1.347 | (0.577) |
| Interpersonal problems | 2224 | 1.918 | (0.321) | 2224 | 1.922 | (0.337) | 2224 | 2.242 | (0.252) | 2224 | 2.072 | (0.303) | 2224 | 2.005 | (0.424) |
| Fibrinogen | 2224 | 1.103 | (0.169) | 2224 | 1.078 | (0.148) | 2224 | 1.307 | (0.182) | 2224 | 1.111 | (0.185) | 2224 | 1.197 | (0.316) |
| CRP | 2224 | 1.352 | (0.234) | 2224 | 1.286 | (0.256) | 2224 | 1.835 | (0.226) | 2224 | 1.528 | (0.268) | 2224 | 1.302 | (0.217) |
| Glucose | 2224 | 1.067 | (0.096) | 2224 | 1.066 | (0.101) | 2224 | 1.086 | (0.077) | 2224 | 1.136 | (0.168) | 2224 | 1.093 | (0.130) |
| Insulin | 2224 | 1.203 | (0.197) | 2224 | 1.190 | (0.174) | 2224 | 1.145 | (0.158) | 2224 | 1.203 | (0.196) | 2224 | 1.238 | (0.167) |
| Triglycerides | 2224 | 2.140 | (0.403) | 2224 | 2.156 | (0.428) | 2224 | 2.165 | (0.483) | 2224 | 2.215 | (0.446) | 2224 | 2.305 | (0.481) |
| LDL | 2224 | 2.056 | (0.458) | 2224 | 2.030 | (0.457) | 2224 | 2.251 | (0.412) | 2224 | 2.018 | (0.386) | 2224 | 2.084 | (0.484) |
| HDL | 2224 | 2.081 | (0.729) | 2224 | 2.081 | (0.729) | 2224 | 2.081 | (0.729) | 2224 | 2.081 | (0.729) | 2224 | 2.081 | (0.729) |
| FSH | 2224 | 1.256 | (0.280) | 2224 | 1.328 | (0.367) | 2224 | 1.343 | (0.347) | 2224 | 1.436 | (0.367) | 2224 | 1.535 | (0.345) |
| Menopausal stage | |  |  |  |  |  |  |  |  |  |  |  |  |  |  |
| Pre | 1236 | 55.576 | – | 829 | 37.275 | – | 643 | 28.912 | – | 409 | 18.390 | – | 50 | 2.248 | – |
| Early Peri | 988 | 44.424 | – | 1254 | 56.385 | – | 1078 | 48.471 | – | 805 | 36.196 | – | 576 | 25.899 | – |
| Late Peri | – | – | – | 99 | 4.451 | – | 199 | 8.948 | – | 234 | 10.522 | – | 241 | 10.836 | – |
| Post | – | – | – | 42 | 1.888 | – | 304 | 13.669 | – | 776 | 34.892 | – | 1357 | 61.016 | – |
| Estradiol | 2224 | 1.144 | (0.168) | 2224 | 1.239 | (0.302) | 2224 | 1.224 | (0.280) | 2224 | 1.047 | (0.099) | 2224 | 1.098 | (0.199) |
|  |  |  |  |  |  |  |  |  |  |  |  |  |  |  |  |

*Note. M* = mean; *SD* = standard deviation; Min = minimum; Max = maximum.; LDL = low density lipoprotein; HDL = high density lipoprotein; CRP = C-reactive protein; FSH = follicle-stimulating hormone; Pre = Pre-menopausal; Peri = Peri-menopausal; Post = Post-menopausal. All scores have been rescaled to range from 1 to 4.

Table 2

*Cross-Construct Cross-Lagged Directed Edges of Within-Person Temporal (Lag-1) Network*

| Node-Out | Node-In | Edge | *p* | *d* |  | Node-Out | Node-In | Edge | *p* | *d* |
| --- | --- | --- | --- | --- | --- | --- | --- | --- | --- | --- |
| dep | fbr | 0.00197 | 0.000 | 0.081 |  | fbr | dep | -0.00054 | 0.000 | -0.076 |
| dep | crp | 0.00012 | 0.000 | 0.004 |  | **fbr** | **som** | **0.00166** | **0.000** | **0.156** |
| dep | glc | 0.00075 | 0.000 | 0.029 |  | fbr | int | -0.00172 | 0.000 | -0.365 |
| dep | ins | 0.00090 | 0.000 | 0.008 |  | **crp** | **dep** | **0.00081** | **0.000** | **1.072** |
| dep | trg | -0.00091 | 0.000 | -0.009 |  | **crp** | **som** | **0.00110** | **0.000** | **1.812** |
| dep | ldl | 0.00131 | 0.000 | 0.094 |  | **crp** | **int** | **0.00033** | **0.000** | **2.112** |
| dep | hdl | -0.00031 | 0.000 | -0.008 |  | glc | dep | -0.00201 | 0.000 | -0.289 |
| **som** | **fbr** | **0.00388** | **0.000** | **0.188** |  | glc | som | -0.00006 | 0.000 | -0.005 |
| som | crp | 0.00164 | 0.000 | 0.056 |  | glc | int | -0.00345 | 0.000 | -0.715 |
| som | glc | 0.00061 | 0.000 | 0.025 |  | ins | dep | -0.00009 | 0.000 | -0.245 |
| som | ins | 0.00074 | 0.000 | 0.009 |  | ins | som | -0.00135 | 0.000 | -6.245 |
| som | trg | 0.00064 | 0.000 | 0.006 |  | ins | int | -0.00033 | 0.000 | -0.708 |
| som | ldl | 0.00087 | 0.000 | 0.099 |  | trg | dep | 0.00000 | 0.000 | -0.004 |
| som | hdl | 0.00157 | 0.000 | 0.046 |  | **trg** | **som** | **0.00014** | **0.000** | **0.131** |
| **int** | **fbr** | **0.00284** | **0.000** | **0.129** |  | trg | int | 0.00007 | 0.000 | 0.071 |
| int | crp | -0.00159 | 0.000 | -0.058 |  | **ldl** | **dep** | **0.00155** | **0.000** | **0.251** |
| int | glc | -0.00086 | 0.000 | -0.036 |  | ldl | som | -0.00043 | 0.000 | -0.074 |
| int | ins | -0.00096 | 0.000 | -0.006 |  | **ldl** | **int** | **0.00101** | **0.000** | **0.327** |
| int | trg | -0.00105 | 0.000 | -0.011 |  | **hdl** | **dep** | **0.00172** | **0.000** | **0.196** |
| **int** | **ldl** | **0.00236** | **0.000** | **0.331** |  | **hdl** | **som** | **0.00214** | **0.000** | **0.162** |
| int | hdl | 0.00009 | 0.000 | 0.003 |  | **hdl** | **int** | **0.00082** | **0.000** | **0.134** |
|  |  |  |  |  |  |  |  |  |  |  |

*Note.* crp = C-reactive protein; dep = depressed mood; fbr = fibrinogen; glc = fasting glucose; hdl = high density lipoprotein; ins = insulin; int = interpersonal problems; lip = lipid marker composite; ldl = low density lipoprotein; som = somatic symptoms; trg = triglycerides. Bold values reflect statistically significant cross-construct edges.

Figure 1

*Within-Person Temporal Network of Proinflammatory Proteins, Lipid Markers, and Depression Nodes*

*Note.* crp = C-reactive protein; dep = depressed mood; fbr = fibrinogen; glc = fasting glucose; hdl = high density lipoprotein; ins = insulin; int = interpersonal problems; lip = lipid marker composite; ldl = low density lipoprotein; som = somatic symptoms; trg = triglycerides. Blue bold lines indicate statistically significant positive relations, whereas red dotted lines signal statistically significant negative relations, and line boldness and thickness reflect strength of associations.

Figure 2

*Within-Person Contemporaneous Network of Proinflammatory Proteins, Lipid Markers, and Depression Nodes*

*Note.* crp = C-reactive protein; dep = depressed mood; fbr = fibrinogen; glc = fasting glucose; hdl = high density lipoprotein; ins = insulin; int = interpersonal problems; lip = lipid marker composite; ldl = low density lipoprotein; som = somatic symptoms; trg = triglycerides. Blue bold lines indicate statistically significant positive relations, whereas red dotted lines signal statistically significant negative relations, and line boldness and thickness reflect strength of associations.

Figure 3

*Between-Person Network of Proinflammatory Proteins, Lipid Markers, and Depression Nodes*

*Note.* crp = C-reactive protein; dep = depressed mood; fbr = fibrinogen; glc = fasting glucose; hdl = high density lipoprotein; ins = insulin; int = interpersonal problems; lip = lipid marker composite; ldl = low density lipoprotein; som = somatic symptoms; trg = triglycerides. Blue bold lines indicate statistically significant positive relations, whereas red dotted lines signal statistically significant negative relations, and line boldness and thickness reflect strength of associations.

1. Due to space constraints, we offer more details on the procedures, measures, and statistical analyses in Appendix A of the OSM. [↑](#footnote-ref-2)
